# Synthetic Biology Tool Development Advances Predictable Gene Expression in the Metabolically Versatile Soil Bacterium *Rhodopseudomonas palustris*

**DOI:** 10.3389/fbioe.2022.800734

**Published:** 2022-03-16

**Authors:** Cheryl M. Immethun, Mark Kathol, Taity Changa, Rajib Saha

**Affiliations:** Department of Chemical and Biomolecular Engineering, University of Nebraska-Lincoln, Lincoln, NE, United States

**Keywords:** synthetic biology tools, non-model microorganism, predictable gene expression, plasmid stability, intrinsic antibiotic resistance

## Abstract

Harnessing the unique biochemical capabilities of non-model microorganisms would expand the array of biomanufacturing substrates, process conditions, and products. There are non-model microorganisms that fix nitrogen and carbon dioxide, derive energy from light, catabolize methane and lignin-derived aromatics, are tolerant to physiochemical stresses and harsh environmental conditions, store lipids in large quantities, and produce hydrogen. Model microorganisms often only break down simple sugars and require low stress conditions, but they have been engineered for the sustainable manufacture of numerous products, such as fragrances, pharmaceuticals, cosmetics, surfactants, and specialty chemicals, often by using tools from synthetic biology. Transferring complex pathways has proven to be exceedingly difficult, as the cofactors, cellular conditions, and energy sources necessary for this pathway to function may not be present in the host organism. Utilization of unique biochemical capabilities could also be achieved by engineering the host; although, synthetic biology tools developed for model microbes often do not perform as designed in other microorganisms. The metabolically versatile *Rhodopseudomonas palustris* CGA009, a purple non-sulfur bacterium, catabolizes aromatic compounds derived from lignin in both aerobic and anaerobic conditions and can use light, inorganic, and organic compounds for its source of energy. *R. palustris* utilizes three nitrogenase isozymes to fulfill its nitrogen requirements while also generating hydrogen. Furthermore, the bacterium produces two forms of RuBisCo in response to carbon dioxide/bicarbonate availability. While this potential chassis harbors many beneficial traits, stable heterologous gene expression has been problematic due to its intrinsic resistance to many antibiotics and the lack of synthetic biology parts investigated in this microbe. To address these problems, we have characterized gene expression and plasmid maintenance for different selection markers, started a synthetic biology toolbox specifically for the photosynthetic *R. palustris*, including origins of replication, fluorescent reporters, terminators, and 5′ untranslated regions, and employed the microbe’s endogenous plasmid for exogenous protein production. This work provides essential synthetic biology tools for engineering *R. palustris*’ many unique biochemical processes and has helped define the principles for expressing heterologous genes in this promising microbe through a methodology that could be applied to other non-model microorganisms.

## Introduction

Biomanufacturing processes that utilize microorganisms can produce specialty chemicals without toxic byproducts that have a deleterious effect on the environment. These processes often only require relatively simple reactions schemes while creating complex and chiral compounds (Wang and Weller, 2006; Engels et al., 2008; Ajikumar et al., 2010; Sasidharan et al., 2011). Some of these processes utilize the unique biochemical capabilities of non-model bacteria (Basak and Das, 2007; Yan and Fong, 2017; Liang et al., 2020). For instance, various *Geobacter* species can produce bioelectricity (Lovley et al., 2011; Rosenbaum and Henrich, 2014). *Burkholderia sacchari* (Guamán et al., 2018), *Psuedomonas spp*., and *Halomonas spp*. (Zhang et al., 2020) synthesize polyhydroxyalkanoates (PHAs), sustainable alternatives to petroleum-derived plastics. Many purple non-sulfur bacteria can also produce PHAs as well as hydrogen, and catabolize a wide array of substrates that are commonly considered waste products (Basak and Das, 2007; Özgür et al., 2010; Brown et al., 2020; Alsiyabi et al., 2021).

Model organisms like *Escherichia coli* are most commonly used in biochemical processes due to their rapid growth rate and comprehensive characterization. *E. coli* has been engineered to produce a number of value-added chemicals, including ethanol (Yazdani and Gonzalez, 2008), 1-butanol (Atsumi et al., 2008) methyl-ketone (Wang et al., 2018), a styrene monomer (Liang et al., 2020), and various pharmaceuticals from organic feedstocks (Chang and Keasling, 2006; Kizer et al., 2008). However, many attempts to express heterologous pathways in model bacteria have proven to be fraught with difficulties. In addition to the substantial engineering work required to introduce all of the edits into the genome, synthesis of all of the new proteins can cause a severe increase in the host’s metabolic load and subsequent growth deficits (Kizer et al., 2008; Müller et al., 2015). Furthermore, the new pathway can produce metabolites that are toxic to non-native host (Chang and Keasling, 2006; Kizer et al., 2008; Nowroozi et al., 2014; Müller et al., 2015; Ng et al., 2015). Model microorganisms can also lack the necessary metabolites to synthesize the desired products (Müller et al., 2015; Wu et al., 2018). In addition, the pathway can be imbalanced in the new host, leading to a shortage of required enzymes for crucial reactions (Nowroozi et al., 2014). This can lead to lower product yields than what was found in the native organism (Wu et al., 2018). Engineering the native host to harness its vast metabolic abilities as the natural producer of the value-added chemical(s) could address these issues.

To harness the large metabolic potential of non-model bacteria, it is vital to build tools that enable precise and predictable gene expression in these microorganisms. Synthetic biology toolkits have been developed for non-model organisms such as cyanobacteria in order to increase titers of value-added chemicals these organisms produce (Markley et al., 2015; Nozzi et al., 2017; Mukherjee et al., 2020). Cyanobacteria’s ability to conduct photosynthesis has been utilized for the production of chemicals such as ethanol (Namakoshi et al., 2016), ethylene (Wang et al., 2018), and isoprene (Bentley et al., 2014). Toolkits have also been developed for thermophiles for the production of organic acids and biofuels like ethanol, hydrogen, and butanol (Zeldes et al., 2015). These toolkits included methods for altering gene expression in non-model organisms such as RBS optimization (Ng et al., 2015; Nieuwkoop et al., 2019), organism specific promoter and terminator libraries (Du et al., 2012; Elmore et al., 2017), and context optimization, since the order of the genes affects transcription rates (Smanski et al., 2014). Despite this progress, the development of synthetic biology tools for non-model organisms is still limited, as the performance of genetic parts are often organism specific (Martínez-García and de Lorenzo, 2017; Yan and Fong, 2017).

*Rhodopseudomonas palustris* CGA009 (hereafter *R. palustris*) is a purple non-sulfur bacterium (PNSB) capable of all four modes of metabolism. *R. palustris*’ chemotrophic and phototrophic abilities provide the energy necessary for energy-intensive biochemical reactions, such as nitrogen and carbon fixation (Larimer et al., 2004). *R. palustris* can catabolize a variety of carbon sources, including a vast array of aromatic compounds such as lignin breakdown products (Harwood and Gibson, 1988; Ramasubramanian et al., 1996; Barbosa et al., 2001; Austin et al., 2015). It can also produce polyhydroxybuturate (Brown et al., 2020) and hydrogen (Barbosa et al., 2001; Huang et al., 2010), and can store up to 39% of its biomass as fatty acids that can be used in biofuels (Carlozzi et al., 2010). *R. palustris* has also gained interest as a tool for bioremediation as it possesses the ability to remove nutrients from wastewater to within European standards (Cerruti et al., 2020). All of *R. palustris*’ beneficial traits make the metabolically robust organism an ideal candidate as a biotechnology chassis. Extensive research has been done on the characterization and improvement of the hydrogen production (Gosse et al., 2010; McKinlay et al., 2014) and the PHB production capabilities of *R. palustris* (Ranaivoarisoa et al., 2019; Alsiyabi et al., 2021). In addition, *R. palustris* has been engineered to produce n-butanol (Doud et al., 2017; Bai et al., 2021). Despite the engineering interest in *R. palustris*, their success has been limited.

Several difficulties inhibit the use of *R. palustris* as a biocatalyst. Engineering efforts with *R. palustris* struggle with its natural resistance to antibiotics (Larimer et al., 2004), requiring high concentrations to maintain selective conditions (Welander et al., 2012; Xu et al., 2016). Transient expression of genes expressed from non-native plasmids has also been a problem (Du Toit et al., 2021). In addition, genetic parts essential for engineering the bacterium have not been tested in *R. palustris.* Fluorescent reporters enable *in vivo* characterization of synthetic biology parts; yet, the background fluorescence can complicate their use in pigmented bacteria like *R. palustris*. The performance of basic building blocks of gene expression, such as origins of replication, 5′ untranslated regions (UTRs), and transcriptional terminators, has also not been determined for this microbe, thus limiting the biotechnology applications. This study tackles these problems by characterizing the bacterium’s *“famous”* insensitivity to antibiotic selection, the stability of exogenous plasmids, and the performance of fluorescent reporters. In addition, two transcriptional terminators are tested to minimize the effect of genetic context, and design rules for 5′ UTRs are explored. To improve the maintenance of heterologous gene expression in *R. palustris,* an expression cassette was then integrated into three sites in the bacterium’s endogenous plasmid. Adding these tools for producing heterologous proteins and clarifying their limitations in *R. palustris* advances the efforts to engineer this robust microbe.

## Materials and Methods

### Strain Growth Conditions

*Rhodopseudomonas palustris* (Molisch) van Niel BAA-98, strain designation CGA009 (*R. palustris*), was obtained from American Type Culture Collection. NEB^®^ 10-beta competent *Escherichia coli* (*E. coli* DH10β) was used for plasmid construction. All strains used in this study are described in Supplementary Table SI, and were stored at −80°C, *R. palustris* strains were stored with a final concentration of 20% glycerol while *E. coli* strains were stored at a 15% final glycerol concentration. Before growth in liquid media, *E. coli* and *R. palustris* strains were grown on solid LB media (Miller, AMRESCO) and 112 Van Niel’s (VN) media (ATCC) (1% yeast extract, 0.1% K_2_HPO_4,_ 0.05% MgSO_4,_ pH 7.1) plates respectively with the appropriate antibiotic (listed below). All strains were grown in 4 ml of their respective media in 14 ml BD Falcon™ round-bottom polystyrene tubes at 275 rpm in the dark in ambient air at 30°C. *E. coli* cultures containing plasmids with ampicillin (amp), gentamicin (gent), or kanamycin (kan) selection markers were grown with 100 μg/ml amp, 10 μg/ml gent, or 30 μg/ml kan, respectively. *R. palustris* cultures were grown in 112 Van Niel’s (VN) media (ATCC) or photosynthetic media (PM) (Brown et al., 2020) supplemented with 20 mM NaC_2_H_3_O_2_, 10 mM NaHCO_3_, and 15.2 mM (NH_4_)_2_SO_4_ where specified. *R. palustris* cultures containing plasmids with the corresponding selection markers were grown with 25, 50, or 100 μg/ml amp, 300 μg/ml gent, or 300 μg/ml kan.

### Strain Construction

Oligonucleotides were purchased from Eurofins Genomics or Integrative DNA Technologies™. All plasmids used in this work are listed in Supplementary Table SI. The sequences of the genetic parts are listed in Supplementary Table SII. PCR was conducted using Phusion Hot Start II DNA Polymerase (Thermo Scientific™). On condition that the PCR product was the desired size as determined through gel electrophoresis with 1X TAE buffer 1% agarose gels, the PCR reaction was purified via Monarch^®^ DNA Gel Extraction Kit (New England Biolabs^®^ Inc.). Plasmids were constructed via the Hot Fusion Assembly Method (Fu et al., 2014) and used to transform *E. coli* DH10β. *E. coli* was grown overnight, diluted to 1/40 OD_600_ in fresh LB media, grown until mid-exponential phase, and washed at room temperature according to published literature (Tu et al., 2016). Washed cells were then transformed by the Hot Fusion product through electroporation. Electroporated cells were incubated at 30°C for 1.5 h in LB media without antibiotic, then plated onto LB plates supplemented with appropriate antibiotic. Plates were incubated overnight at 30°C. Colonies were then selected and cultures were grown in LB media overnight with the appropriate antibiotic. Cultures were stored as 15% (v/v) glycerol stock at −80°C. Plasmids were harvested from the cultures using the PureLink™ Quick Plasmid Miniprep Kit (Invitrogen™). After extraction of the assembled plasmids, junctions in the plasmids were confirmed by submission of PCR products to Eurofins Genomics for DNA sequencing. Plasmids were then used to transform *R. palustris* through the same method with the following exceptions. *R. palustris* was diluted to 0.2 OD_660_ and grown overnight. After electroporation, *R. palustris* was incubated in VN media without antibiotic overnight, then plated onto VN plates supplemented with appropriate antibiotic. Plates were incubated approximately 5–7 days after transformation until colonies emerged. These colonies were then streaked onto fresh VN plates supplemented with appropriate antibiotic and allowed to grow approximately 5 days. Liquid cultures were grown from these plates in VN media with appropriate antibiotic. *R. palustris* cultures were stored at −80°C at a final concentration of 20% (v/v) glycerol. The template for Colony PCR was cells suspended in water and lysed at 100°C for 25 min. PCR products of sequences critical for performance were confirmed by submission of PCR products to Eurofins Genomics for DNA sequencing.

Sucrose counter selection was used to remove the chloramphenicol acetyltransferase gene from *R. palustris.* Briefly, a suicide plasmid was constructed using the p15A origin of replication. The plasmid (pΔcat) contained two adjacent 1,300–1,500 bp homology arms to allow for integration into *R. palustris’* genome and are described in Supplementary Table SIII. This plasmid also contained a gentamicin resistance gene to provide selection pressure and a *sacB* gene to confer sucrose lethality. This plasmid was used to transform *R. palustris* following the previously described method and plated onto VN/gentamicin plates (300 μg/ml gent). Gentamicin-resistant colonies were then picked and grown in 4 ml PM with 10 mM sodium succinate without gentamycin. Cultures were grown at 30°C and 275 rpm for 2 days to allow for recombination. Cells were then removed from cultures through centrifugation and resuspended in 200 ml PM media. Serial dilutions of 1/1,000 and 1/10,000 were then performed on these cultures and dilutions were plated on PM media plates supplemented with 10% sucrose. Plates were incubated for 6 days to allow colonies to emerge. Colonies were then tested on duplicate grid plates. This was done by transferring a colony to a PM plate supplemented with 215 μg/ml gentamicin and simultaneously transferring to a plain PM plate to determine a loss of vector-mediated gentamicin-resistance. Colonies that did not grow on PM plates supplemented with gentamicin, but did grow on plain PM media plates were then selected and transferred into culture tubes containing 5 ml PM. Cultures were then incubated at 30°C for 6 days until a purple color appeared. Colony PCR with the segregation primers listed in Supplementary Table IV was performed and sequencing via submission to Eurofins Genomics was used to verify the deletion strain.

Mutations to *R. palustris*’ endogenous plasmid were accomplished through double homologous recombination. Briefly, a suicide plasmid was constructed from the expression cassette flanked by two 800–1,000 bp homology arms and the p15A origin of replication (ori). All homology arm sequences are described in Supplementary Table SIII. Colony PCR was used to validate that the ori does not replicate in *R. palustris*. The homology arms were amplified from *R. palustris* gDNA which was obtained using Monarch^®^ Genomic DNA Purification Kit (New England Biolabs^®^ Inc.). A gentamicin selection marker was included in the expression cassette to force segregation into the endogenous plasmid. Electroporation of the suicide plasmid and culturing of the subsequent colonies was conducted following the previously described methods. Colony PCR with the segregation primers listed in Supplementary Table SIV was performed and sequencing via submission to Eurofins Genomics was used to verify the incorporation of the expression cassette.

### Aerobic Antibiotic Tolerance Determination

Wild type *R. palustris* cultures were grown in 50 ml of PM media supplemented with 20 mM NaC_2_H_3_O_2_, 10 mM NaHCO_3_, 15.2 mM (NH_4_)_2_SO_4_, in 250 ml Erlenmeyer flasks to stationary phase. Cultures were then diluted to 0.2 OD_660_ into 70 ml of fresh PM media supplemented with 20 mM NaC_2_H_3_O_2_, 10 mM NaHCO_3_, 15.2 mM (NH_4_)_2_SO_4_, and one of the following antibiotics; 50, 100 μg/ml amp, 34 μg/mL cm, 300 ug/mL gent, 200, 300 μg/ml kan, or 300 μg/ml spec. The cultures were then transferred into glass 85 ml tubes, and placed into a Multi-Cultivator 1000-OD (Photon Systems Instruments). Theses cultures were incubated at 30°C, in the dark, and bubbled with ambient air. The cultures’ absorbance (680 nm) was measured by the Multi-Cultivator 1000-OD every 2 h until it did not change for at least 3 measurements.

### Anaerobic Antibiotic Tolerance Determination

Wild type *R. palustris* cultures were aerobically grown in 50 ml of PM media supplemented with 20 mM NaC_2_H_3_O_2_, 10 mM NaHCO_3_, 15.2 mM (NH_4_)_2_SO_4_, in 250 ml Erlenmeyer flasks to stationary phase. Cultures were then diluted to 0.2 OD_660_ into fresh PM media supplemented with 20 mM NaC_2_H_3_O_2_, 10 mM NaHCO_3_, 15.2 mM (NH_4_)_2_SO_4_, and one of the following antibiotics; 50 μg/ml amp, 300 ug/mL gent, or 300 μg/ml kan, in addition to the no antibiotic control. 14 ml BD Falcon™ round-bottom polystyrene tubes were filled and sealed in triplicate for each culture and incubated at 30°C, 275 rpm, in 100 µE white light for 1 week. These replicates were then streaked onto PM plates, no antibiotics for any culture, and allowed to grow in ambient air at 30°C for 10 days.

### Fluorescence Fold Change Measurement

*R. palustris* strains were grown to stationary phase in PM media supplemented with 20 mM NaC_2_H_3_O_2_, 10 mM NaHCO_3_, 15.2 mM (NH_4_)_2_SO_4_ and the appropriate antibiotic in 250 ml Erlenmeyer flasks before dilution to 0.2 OD_660_ in 500 μL of fresh media and the appropriate antibiotic concentration, in triplicate. The cultures were loaded into a Greiner CELLSTAR^®^ bio-one sterile 48 well culture plate and covered with a Breathe-Easy^®^ Gas Permeable Sealing Membrane (Diversified Biotech). Cultures were then incubated in the dark at 30°C in ambient air for 72 h at 275 rpm. Afterwards, 200 μL of each culture was pipetted into a Greiner bio-one 96 well polystyrene flat-bottomed μCLEAR^®^ black microplate before fluorescence of reporter proteins and absorbance of the cultures were measured. Three wells were also loaded with PM media to act as blanks. Plate was then loaded into a Molecular Devices SpectraMax^®^ i3x microplate reader. Excitation-Emission wavelengths for mRFP, eYFP, and GFPuv were respectively: 583–608 nm, 485–528 nm, and 395–509 nm. Absorbance of cultures at 660 nm was also measured. The following equation was used to calculate each strain’s relative fluorescence.

$$Fluorescent\;fold\;change={\left(\frac{\left(\frac{\left(F_{mrfp}-F_{media}\right)}{\left(A_{mrfp}-A_{media}\right)}\right)}{{\left(\frac{\left(F_{wt}-F_{media}\right)}{\left(A_{wt}-A_{media}\right)}\right)}_{avg}}\right)}_{avg}$$

F_mrfp_ Fluorescence of mRFP-producing culture.

F_media_ Average fluorescence of media blanks.

F_wt_ Fluorescence of wild type culture.

A_mrfp_ Absorbance (OD_660_) of mRFP-producing culture.

A_media_ Absorbance (OD_660_) of media blanks.

A_mrfp_ Absorbance (OD_660_) of wild type culture.

### Flow Cytometry

*R. palustris* strains were grown according the Fluorescent Fold Change Measurement section. Cultures were then resuspended in 150 μL 0.85% NaCl at 0.001 OD_660_ and loaded into a Fisherbrand clear, polystyrene, 350 μL, flat-bottom 96 well plate. The cultures were analyzed by a Beckman Coulter CytoFLEX LX flow cytometer. mRFP was excited by a 561 nm yellow/green laser and the emission was collected using a 610/20 nm bandpass filter. 10,000 bacterial events were collected per sample.

### β-Galactosidase Assay

*R. palustris* strains were grown according to the Fluorescent Fold Change Measurement section. 200 μL of each culture was then pipetted into a Greiner bio-one 96 well polystyrene flat-bottomed μCLEAR^®^ black microplate and absorbance (660 nm) of the cultures was measured. Lysis of the cells and the measurement of β-Galactosidase activity followed a previously published method (Thibodeau et al., 2004), except that 5 mM 1,4-dithiothreitol was added to the Z buffer instead of β-mercaptoethanol. A Molecular Devices SpectraMax^®^ i3x microplate reader was used to measure the absorbance of the intact cells (660 nm), and the lysed cells treated with ONPG (o-nitrophenyl-β-D-galactopyranoside) over time. LacZ can convert ONPG to o-nitrophenol, which is yellow in color and can be measured at 420 nm. The increase in yellow color (absorbance at 420 nm) was normalized to the absorbance measured at 660 nm.

### Construction of the RBS Library and 5’ UTR Replacement

To construct the Ribosome Binding (RBS) library, *mrfp*’s six base pair RBS was subjected to saturation mutagenesis using degenerate oligonucleotides for PCR. The Blunt End Ligation Method was used to construct the plasmid with T4 DNA Ligase and T4 Polynucleotide Kinase (New England Biolabs). The sequence of the RBS of selected colonies was determined by submission of colony PCR products to Eurofins Genomics for DNA sequencing (Supplementary Table SV). The De Novo DNA RBS calculator was used to predict the translation initiation rate of the *mrfp* 5′ UTRs (Salis et al., 2009; Borujeni et al., 2014; Borujeni and Salis, 2016; Borujeni et al., 2017; Reis and Salis, 2020; Cetnar and Salis, 2021). The De Novo DNA RBS calculator for controlling translation initiation rates was then used to design the 5′ UTR sequences for the eYFP plasmids. The 5′ UTRs were exchanged using Blunt End Ligation method. All 5′ UTR sequences are listed in Supplementary Table SII.

### RNA Extraction, gDNA Removal, and Reverse Transcription

RNA was extracted from *R. palustris* cultures at mid-exponential phase in duplicate according to TRIzol^®^ Reagent (Life Technologies™) protocol, with the following modifications. After the addition of chloroform required for phase separation, cells were incubated at room temperature for 5 min. After incubation of samples with isopropanol to precipitate the RNA, cells were centrifuged for 30 min instead of 10 min at 12000 g and 4°C. Ice cold 75% ethanol was used to wash RNA pellets. After the RNA wash, samples were centrifuged for 10 min at 7500 g and 4°C.

RNA extracts were treated with TURBO™ DNase (Invitrogen™) to remove any gDNA that may have also been extracted. Afterwards, PCR of the RNA with the 16SrRNA primers (Supplementary Table SVI) and gel electrophoresis with a 2% agarose gel was used to confirm the absence of any gDNA. A bleach gel was then used to verify the integrity and lack of degradation of RNA after extraction, as described in literature (Aranda et al., 2012). Briefly, 1 μg total RNA was run in a 1X TAE 1% agarose gel with 0.5% (v/v) bleach. Gel electrophoresis was conducted and gels were imaged to verify RNA integrity. Wells that contained two equivalent bands after DNase treatment were considered to not be degraded. RNA samples that were not degraded were converted to cDNA in 20 µL reactions using a High-Capacity cDNA Reverse Transcription Kit (Applied Biosystems™).

### RT-qPCR

All oligonucleotides used for RT-qPCR are outlined in Supplementary Table SVI. Primer concentrations for RT-qPCR reactions were initially evaluated by performing 25 μL PCR reactions using Go Taq^®^ Master Mix (Promega Corporation) with approximately 100 μg of gDNA, for primer concentrations ranging from 350 to 50 nM. A second set of reactions containing no gDNA was also prepared. Thermo cycler settings for this reaction were 95°C for 2 min, 40 cycles of (95°C for 45 s, 60°C for 45 s, 72 for X seconds—based on amplicon length), and then 72°C for 5 min. Gel electrophoresis was then performed on PCR products using 1X TAE 2% agarose gels. These reaction sets were used to determine primer concentrations that both produce a band of the correct size in the reaction set containing gDNA and fail to produce a band in the reaction set without gDNA. Primer concentrations that met these criteria were used to inform primer efficiency tests, thus reducing the consumption of valuable cDNA.

Primer efficiency was determined by preparing qPCR reactions with PowerUp SYBR^®^ Green Master Mix (Life Technologies™), with a 5x dilution series of cDNA (500 ng/μL–0.0061 ng/μL). The primer concentration for each target gene for qPCR reactions is also listed in Supplementary Table SVI. qPCR reactions were run in an Eppendorf Mastercycler Realplex. Thermocycler settings for qPCR reactions were 50°C for 2 min, 95°C for 2 min, 40 cycles of (95°C for 15 s, 60°C for 1 min), with a melting curve step (60°C for 1 min, rising at a rate of 1.5°C for 20 min, staying at 95°C for 15 s) to ensure a single amplicon. Non-template controls (NTC) in triplicate were included for each reaction to ensure a lack of unintended PCR products for each target gene. The Eppendorf Mastercycler Realplex automatic baseline calculator was used to calculate the baseline for samples. Cycle threshold (C_T_) values were then obtained from the Eppendorf Mastercycler Realplex. Primer efficiency for each primer set reaction was determined by plotting the Log_10_ of cDNA copies vs. the C_T_ value for each set of reactions. Linear regression was used to ensure linearity of this plot. The slope of this graph determined the primer efficiency of the reaction. The ThermoFisher Scientific qPCR calculator was used to determine primer efficiency for each reaction set. Using this method, all primers were ensured to have a primer efficiency between 90 and 110%., reported in Supplementary Table SVI.

qPCR reactions for two biological replicates and two technical replicates were then run in the Eppendorf Mastercycler Realplex using the same settings as for primer efficiency determination. All qPCR reactions were performed using PowerUp SYBR^®^ Green Master Mix (Life Technologies™). The concentrations of primers and cDNA are listed in Supplementary Table SVI. NTC controls in triplicate were also run. The relative mRNA concentration was calculated per the following equation, GOI is the gene of interest and HK is the housekeeping gene.

$$\mathrm{Relative}\;\mathrm{mRNA}\;\mathrm{concentration}={\left(2^{-\left(C_{T}GOI-C_{T}HK\right)}\right)}_{avg}$$

C_T_GOI C_T_ value of gene of interest.

C_T_HK C_T_ value of housekeeping gene.

### Plasmid Copy Number

qPCR with gDNA was used to determine the absolute plasmid copy number ratio of both native and non-native plasmids relative to the chromosome as described previously (DeLorenzo et al., 2018). gDNA was extracted from *R. palustris* cultures at mid-exponential phase in duplicate using the Monarch^®^ Genomic DNA Purification Kit (New England Biolabs^®^ Inc.). This kit uses alkaline lysis and a silica membrane column, which have been shown to efficiently extract and purify both plasmids and the chromosomes (Becker et al., 2016). Amplified PCR product using primers in Supplementary Table SVI was diluted to 10^9^ copies of amplicon/μL per the following equation.

$$\mathrm{Amplicon}\;\mathrm{copy}\;\mathrm{number}=\frac{\left(6.02\;x10^{23}\frac{copies}{mol}\right)\left(gDNA\frac{ng}{µL}\right)\;}{\left(\mathrm{amplicon}\;\mathrm{length}\;\left(bp\right)\right)\left(660\frac{g}{\left(mol\cdot bp\right)}\right)}$$

An external standard curve was constructed using a 5x dilution series of this diluted PCR product, starting with 10^7^ copies/uL in triplicate. The primer efficiency was determined as outlined in the previous section.

qPCR reactions for two biological replicates and two technical replicates were then run in the Eppendorf Mastercycler Realplex using the same settings as for primer efficiency determination. The concentrations of primers and gDNA are listed in Supplementary Table SVI. NTC controls in triplicate were also run. The standard curve was used to determine the number of copies of each amplicon. The plasmid copy number relative to the chromosome was then determined.

$$\mathrm{Plasmid}\;\mathrm{copy}\;\mathrm{number}\;\mathrm{ratio}=\frac{\mathrm{Copies}\;\mathrm{of}\;\mathrm{gene}\;\mathrm{from}\;\mathrm{plasmid}}{\mathrm{Copies}\;\mathrm{of}\;\mathrm{single}\;\mathrm{copy}\;\mathrm{gene}\;\mathrm{in}\;\mathrm{chromosome}}$$

### Statistical Methods

All statistical analyses (Student’s two-tailed *t*-test with unequal variances) were performed using Microsoft Excel. *p*-value < 0.05 was considered to be significant. All experiments were conducted in biological triplicate, with the exception of qPCR and plasmid copy number, which was performed in biological duplicate and technical duplicate. All error bars represent the population standard deviation and were calculated using Excel.

## Results

### Sensitivity to Antibiotics Used for Selection

Engineering *R. palustris* to explore its unique biochemical abilities has required very high concentrations of the antibiotic used for selection (Braatsch et al., 2006; Rey et al., 2006; Huang et al., 2010; Pechter et al., 2016; Doud et al., 2017); yet, characterization of *R. palustris*’ sensitivity to the antibiotics commonly used for genetic engineering was not found in literature searches. To address this problem, either ampicillin, kanamycin sulfate, gentamicin sulfate, or spectinomycin sulfate was added to wild type *R. palustris*’ media and the absorbance (680 nm) was recorded every 2 hours. These antibiotics were chosen since they are commonly used in synthetic biology or have been employed for selection in *R. palustris* previously (Crosby et al., 2010; Yang & Lee, 2011; Pechter et al., 2016). As this bacterium is also phototrophic, antibiotics that are light-sensitive, such as tetracycline, were not considered.

**FIGURE 1:**
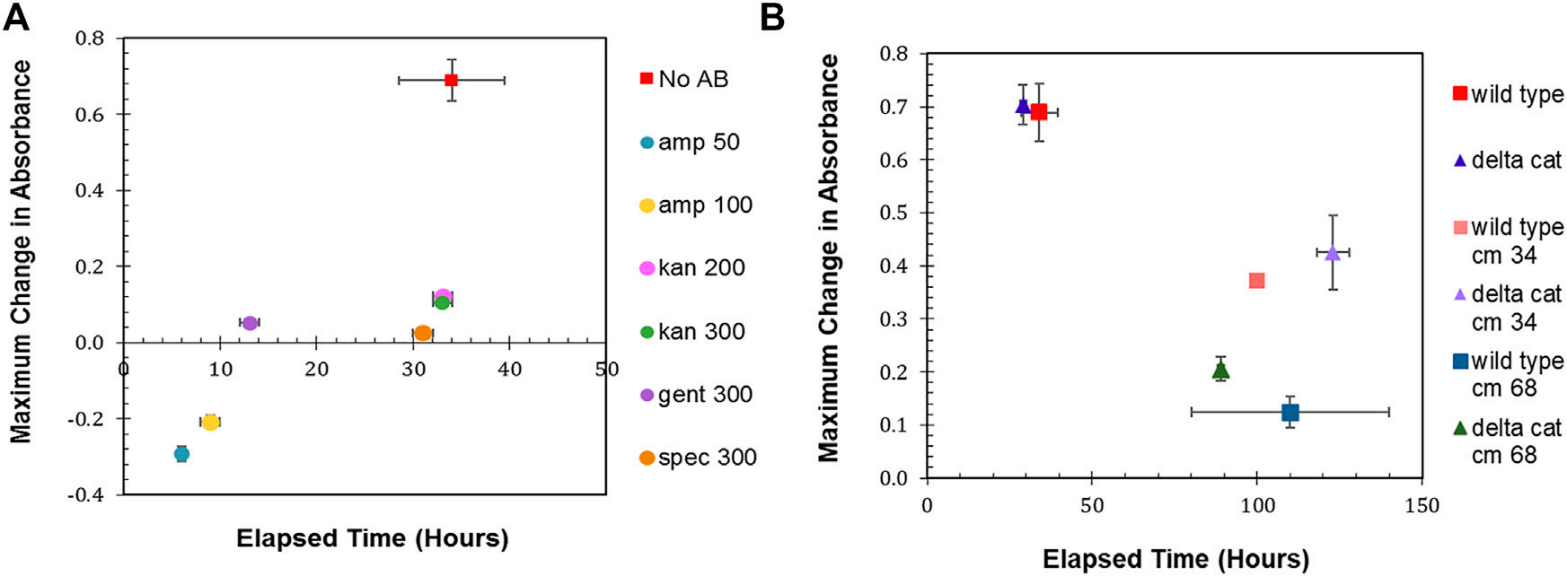
Antibiotic sensitivity. **(A)** Wild type *R. palustris*’ change in absorbance in response to the addition of antibiotics (commonly used in synthetic biology) to the media and growth recorded over time. “No AB” is wild type *R. palustris* without any antibiotics added to the media. Amp 50 and Amp 100 are 50 μg/ml and 100 μg/ml ampicillin respectively. Kan 200 and Kan 300 are 200 μg/ml and 300 μg/ml kanamycin sulfate respectively. Gent 300 and Spec 300 are 300 μg/ml gentamicin sulfate and 300 μg/ml spectinomycin sulfate respectively. **(B)** Change in absorbance for wild type *R. palustris* and the *R. palustris* Δ*cat* strain in response to the addition of 34 and 68 μg/ml chloramphenicol added to the media for both strains and growth recorded over time.

The maximum change in absorbance and the time to achieve that maximum change was then determined (Figure 1A). *R. palustris* was most sensitive to ampicillin, with both 50 μg/ml and 100 μg/ml eliminating the bacterium within 10 hours. Kanamycin selection has been commonly used when engineering *R. palutris*; yet, it took over 30 h for the growth (average change in absorbance of 0.1) to completely stop after the addition of either 200 μg/ml or 300 μg/ml. The cultures without any antibiotics reached stationary phase in a similar amount of time. It was also more than 30 h for the absorbance of the cultures with 300 μg/ml spectinomycin to stop changing; although, the average maximum change was only 0.03. Gentamicin has also been used for selection to engineer *R. palustris* (Hirakawa et al., 2012). Adding 300 μg/ml to the *R. palustris* cultures arrested growth in an average of 13 h while the absorbance increased by just 0.05. The growth curves used to determine the maximum change in absorbance and the time to achieve maximum change can be found in the Supplemental Material (Supplementary Figure S1).

In addition to the characterization of *R. palustris’* sensitivity to antibiotics during aerobic growth, 300 μg/ml kanamycin, 300 μg/ml gentamicin, and 50 μg/ml ampicillin were tested for their ability to prevent growth in anaerobic conditions with 100 µE white light for 1 week. The cultures with ampicillin grew equivalently to the cultures without any added antibiotics. Both kanamycin and gentamicin prevented growth, but the cultures maintained a faint pink color that suggested the presence of *R. palustris*. Therefore, these cultures were streaked onto plates without any antibiotics and allowed to grow for 10 days. The cultures that had grown in gentamicin did not produce any growth on the plate, while the cultures that had grown in kanamycin produced multiple single colonies.

A chloramphenicol acetyltransferase (CAT), which inactivates chloramphenicol, is encoded in *R. palustris*’ chromosome. Furthermore, *cat* is expressed in both aerobic and anaerobic conditions (Supplementary Figure S2) as verified by PCR of the cDNA (Materials and Methods). In an effort make *R. palustris* sensitive to chloramphenicol, *cat* was removed from the chromosome using sucrose counterselection (Materials and Methods). Wild type and Δ*cat R. palustris*’ sensitivity to chloramphenicol was then tested with two different concentrations of chloramphenicol, following the same protocol used to test *R. palustris*’ sensitivity to the other antibiotics. Both strains’ growth was hampered by the antibiotic. At 34 μg/ml, the maximum absorbance of both strains was 50% of the same strains grown without chloramphenicol (Figure 1B). At 68 μg/ml, the wild type and Δ*cat* strains only reached 20 and 33% of their maximum growths respectively under a much longer timeframe. This, however still did not constitute adequate selective conditions.

### Reporters for Parts Characterization

**FIGURE 2:**
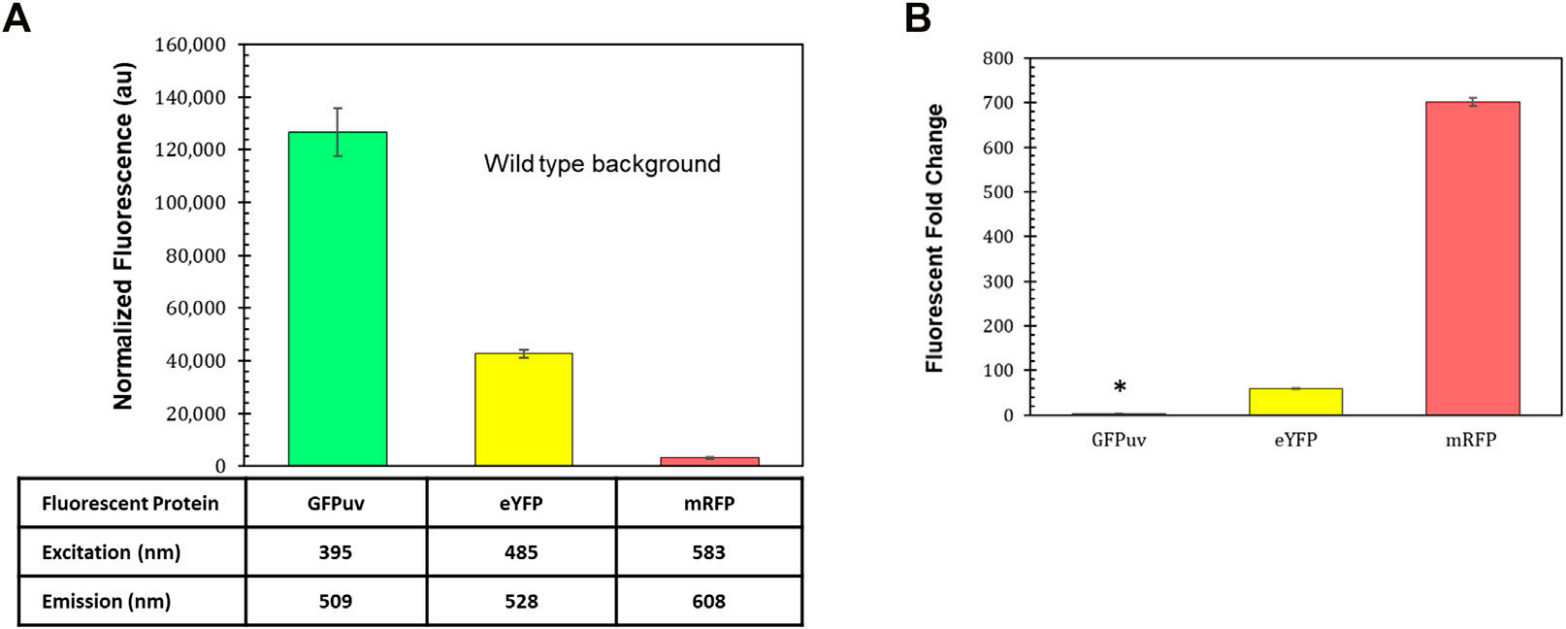
Fluorescent reporters and background fluorescence. **(A)** Average fluorescence of wild type *R. palustris* normalized to absorbance (660 nm), in triplicate, at the emission and excitation wavelengths for GFPuv, eYFP, and mRFP (Materials and Methods). Error bars represent the population standard deviation. **(B)** Fluorescent fold change average of *R. palustris* BBR1-kan-GFPuv, BBR1-kan-eYFP, and BBR1-kan-mRFP strains grown in triplicate. 300 μg/ml kanamycin sulfate was added to the media for the mutant strains but not wild type *R. palustris* (Materials and Methods). * indicates statistically significant expression of 3-fold for the BBR1-kan-GFPuv strain. Error bars represent the population standard deviation.

Fluorescent reporters are a fundamental synthetic biology tool for characterizing how gene expression changes depending on the genetic parts (promoters, terminators, ribosome binding sites, etc.) that are employed (Delvigne et al., 2015). The background fluorescence produced by photosynthetic microbes like *R. palustris* can complicate the use of such an important tool. The gene for green fluorescent protein (GFP) has been expressed in *R. palustris* CGA009 (Doud et al., 2017) as well as another *R. palustris* strain, GJ-22 (Zhai et al., 2019), but the use of other fluorescent proteins has not been reported. To determine the fluorescent reporter best suited for work in *R. palustris*, the fluorescence of wild type *R. palustris* was determined (Materials and Methods) for the excitation and emission wavelengths of GFPuv, enhanced yellow fluorescent protein (eYFP), and monomeric red fluorescent protein (mRFP) (Figure 2A). Interestingly, the pink pigmented bacterium’s background fluorescence was lowest for mRFP. *R. palustris’* background fluorescence was also determined for purple and cyan fluorescent proteins (Supplementary Table SVII). Since this background signal was similar to or higher than GFPuv and eYFP, they were not considered for use as reporters. Next, *R. palustris* transformed by a plasmid using a kanamycin selection marker, the pBBR1 origin of replication (Kovach et al., 1995), and either *gfpuv* (Lee et al., 2011), *eyfp* (Knoot et al., 2019), or *mrfp* (Bi et al., 2013) expressed from the *lac* promoter, and was tested following the protocol for measuring the background fluorescence of the wild type strain. The pBBR1 origin of replication was chosen since it has been used for heterologous gene expression previously in *R. palustris* (Braatsch et al., 2006; Huang et al., 2010; Heiniger and Harwood, 2015). The strain expressing *gfpuv* produced a normalized fluorescence that was just three-fold higher than the wild type strain’s normalized background fluorescence (student’s two-tail *t*-test, *p* < 0.05) while the fluorescent fold change for the *eyfp*-expressing strain was 55 (Figure 2B). To verify the expression of *gfpuv*, RNA was extracted from cultures of the *gfpuv-*expressing strain at mid-exponential phase, in addition to wild type *R. palustris* cultures. PCR reactions were performed on the cDNA obtained from the RNA extracts. These reactions show expression just from the BBR1-kan-GFPuv cultures (Supplementary Figure S2). The *mrfp*-expressing strain produced more than 700-fold higher fluorescence than the wild type background and was thus selected for future work.

The *lacZ* gene from *E. coli* MG1655 was also tested in the BBR1-kan plasmid, replacing *mrfp,*. LacZ hydrolyzes ONPG (o-nitrophenyl-β-D-galactopyranoside) to produce o-nitrophenol, which is a deep yellow color. The absorbance (420 nm) of o-nitrophenol normalized to the absorbance (660 nm) of the cells before lysis (Materials and Methods) quickly rose above the normalized background absorbance from the wild type strain and continued to increase over the hour measured (Supplementary Figure S3).

### Selection Marker Testing

**FIGURE 3:**
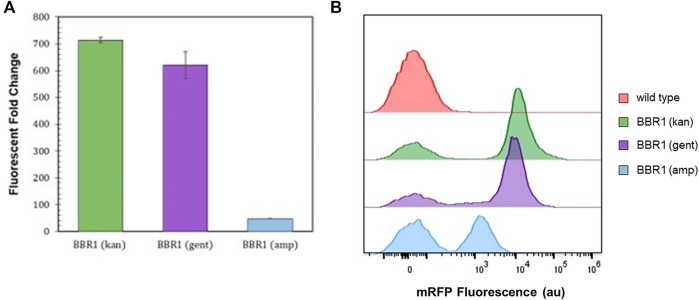
Expression tests and flow cytometry results **(A)** Average fluorescent fold change of *R. palustris* BBR1-kan-mRFP, BBR1-gent-mRFP, and BBR1-amp-mRFP strains grown in triplicate. The antibiotic concentrations added to the media of the mutant strains but not wild type *R. palustris* were 300 μg/ml kanamycin sulfate, 300 μg/ml gentamicin sulfate, and 50 μg/ml ampicillin respectively (Materials and Methods). Error bars represent the population standard deviation. **(B)** Representative flow cytometry results of mRFP fluorescence from *R. palustris* wild type, BBR1-kan-mRFP, BBR1-gent-mRFP, and BBR1-amp-mRFP strains grown in triplicate. The growth procedure, media, and antibiotics are the same as for the fluorescent fold change test in Figure 3A (Materials and Methods).

300 μg/ml kanamycin produced selective conditions that yielded a fluorescent fold change of 700 from the *mrfp-*expressing *R. palustris*. Both ampicillin and gentamicin allowed a smaller positive change in absorbance in a shorter time as compared to kanamycin when testing the microbe’s sensitivity to the antibiotics. Therefore, the kanamycin selection marker (kan) in the BBR1-mRFP plasmid was replaced with an ampicillin resistance gene (amp) or a gentamicin resistance gene (gent) and used to transform *R. palustris.* The fluorescent fold change from the gent strain was statistically similar to the kan strain (student’s two-tail *t*-test, *p* > 0.05) (Figure 3A). When a gentamicin selection marker was used in the BBR1-LacZ plasmid, the normalized absorbance from a β-galactosidase assay with the subsequent BBR1-gent-LacZ strain was almost three times higher after 1 h than for the BBR1-kan-LacZ strain, both tested in triplicate (Supplementary Figure S3).

The fluorescent fold change from the BBR1-amp-mRFP strain produced just a 48-fold change in fluorescence (Figure 3A). Flow cytometry was then employed to investigate the differences in the mRFP-producing strains as compared to the wild type *R. palustris*, all in triplicate (Materials and Methods). All cultures included some cells with just the background fluorescence of the wild type strain. Figure 3B is representative of the results. The averages and standard deviations are presented in Figure 8F. Complete flow cytometry results can be found in Supplementary Figures S7–22. More than 50% of the cells produced just the background fluorescence for the BBR1-amp-mRFP strain with 50 μg/ml amp. This strain was also tested with 25 and 100 μg/ml ampicillin. The average fluorescent fold change was statistically lower (student’s two-tail *t*-test, *p* < 0.05) and the variation was larger when 25 μg/ml ampicillin was used (Supplementary Figure S4). When the ampicillin concentration was increased to 100 μg/ml, all of the cells just produced the wild type background fluorescence (Supplementary Figure S5). For the BBR1-kan-mRFP and BBR1-gent-mRFP strains there were fewer cells with just the background fluorescence, with an average just under 20%. Either a kanamycin or gentamicin selection marker was included in all of the remaining plasmids built for this study.

### Origins of Replication

**FIGURE 4:**
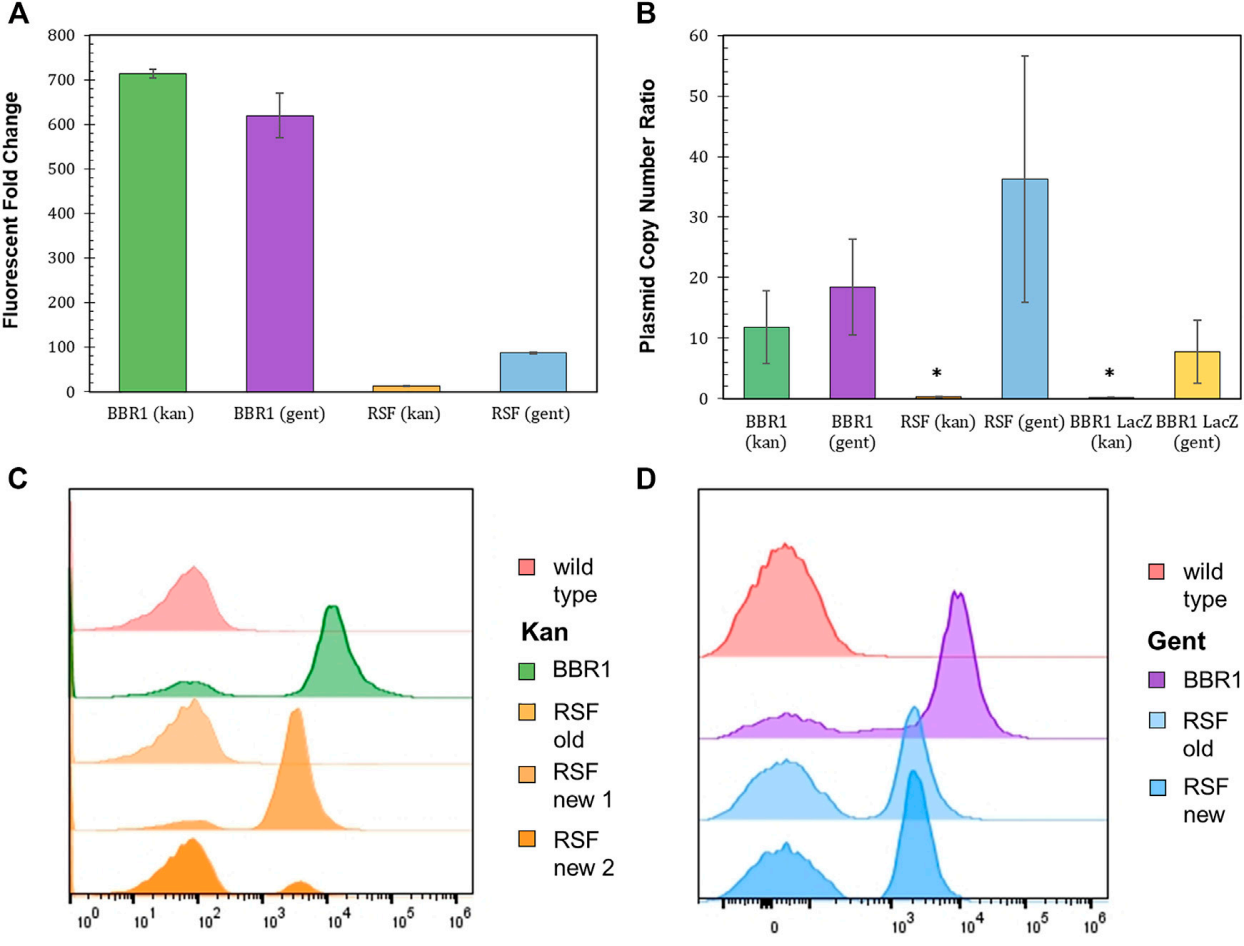
Origin of replication characterization with different selection markers and reporters. **(A)** Fluorescent fold change average of *R. palustris* BBR1-kan-mRFP, BBR1-gent-mRFP, RSF1010-kan-mRFP, and RSF1010-gent-mRFP strains grown in triplicate. The antibiotic concentrations added to the media of the mutant strains but not wild type *R. palustris* were 300 μg/ml kanamycin sulfate and 300 μg/ml gentamicin sulfate respectively (Materials and Methods). Error bars represent the population standard deviation. **(B)** Average plasmid copy number for the *R. palustris* BBR1-kan-mRFP, BBR1-gent-mRFP, RSF1010-kan-mRFP, RSF1010-gent-mRFP, BBR1-kan LacZ, and BBR1-gent-LacZ strains grown in at least duplicate. The antibiotic concentrations added to the media of the mutant strains were 300 μg/ml kanamycin sulfate and 300 μg/ml gentamicin sulfate respectively. Copies of the kanamycin and gentamycin selection marker gene were compared to the single copy *dxs* gene in the chromosome by qPCR and an external standard curve. * indicates a statistically significant plasmid copy number of less than 1, 0.34-fold for the RSF1010-kan-mRFP strain and 0.16 for the BBR1-lan-LacZ strain (Materials and Methods). Error bars represent the population standard deviation. **(C)** Representative flow cytometry results of mRFP fluorescence from *R. palustris* wild type, BBR1-kan-mRFP, and RSF1010-kan-mRFP strains grown in triplicate. The old RSF1010-kan cultures had been streaked from frozen stock 2 weeks earlier. The new RSF1010-kan cultures were single colonies from a new transformation. The growth procedure, media, and antibiotics are the same as for the fluorescent fold change test in Figure 4A (Materials and Methods). **(D)** Representative flow cytometry results of mRFP fluorescence from *R. palustris* wild type, BBR1-gent-mRFP, and RSF1010-gent-mRFP strains grown in triplicate. The old RSF1010-gent cultures had been streaked from frozen stock 2 weeks earlier. The new RSF1010-gent cultures were single colonies from a new transformation. The growth procedure, media, and antibiotics are the same as for the fluorescent fold change test in Figure 4A (Materials and Methods).

Plasmids with the pBBR1 origin of replication (ORI) have been commonly used for heterologous gene expression in *R. palustris* (Braatsch et al., 2006; Huang et al., 2010; Heiniger and Harwood, 2015). Yet, studies that determine how well BBR1 plasmids are maintained by *R. palustris* were not found in literature. Fluorescent fold change, flow cytometry, and the relative quantification of plasmid copy number were utilized to access BBR1’s maintenance under kanamycin or gentamicin selection (Figure 4). Plasmid copy number was of interest because it directly affects the number of proteins produced from the genes encoded on the plasmid, as well the stable distribution of the vector to daughter cells during growth (Jahn et al., 2016). The same tests were also conducted after exchanging the pBBR1 with the RSF1010 replicon, another origin of replication with a broad host range (Taton et al., 2014; Knoot et al., 2019).

As determined when testing the selection markers, the fluorescent fold change for BBR1 plasmids expressing *mrfp* using kan or gent selection were statistically similar, averaging about 650-fold (Figure 4A). There was a significant difference in the fluorescent fold change for the RSF1010 plasmids with the two selection markers, 87-fold for gent and only 12-fold for kan. Flow cytometry of the cultures in triplicate suggest that kanamycin does not maintain enough selection pressure for mRFP to be produced consistently from the RSF1010 plasmid in *R. palustris*. Figure 4C is representative of the results. The averages and standard deviations are presented in Figure 8F. Only cells that produced background fluorescence were found in a RSF1010-kan-mRFP culture that had been streaked from frozen stock 2 weeks earlier. In addition, one culture from a newly transformed RSF1010 colony was predominantly fluorescent cells, 82%, while the number of fluorescent cells in a culture grown from a second colony from the same transformation was just 12%. This variation in cultures harboring the RSF1010 plasmid was not seen when gentamicin was used for selection (Figure 4D). The average number of cells from the RSF1010-gent-mRFP cultures with just background fluorescence was higher than for the BBR1-gent cultures, 50% for RSF1010-gent and 20% for BBR1-gent, but unlike the RSF1010-kan-mRFP cultures, that percentage was not dependent on the culture’s age or whether they had been frozen.

The relative copy number of the BBR1-mRFP, BBR1-LacZ, and RSF1010-mRFP plasmids, with kan and gent selection markers, was determined relative to *R. palustris’* number of chromosomes by qPCR with the cultures’ gDNA and standard curves derived from a dilution series of each PCR product (Materials and Methods) (Lee et al., 2006; DeLorenzo et al., 2018). A section of each selection marker and of a single copy gene in the chromosome were amplified for the comparison. Similar to the fluorescent fold change and flow cytometry results, the relative plasmid number was equivalent for the BBR1-mRFP strains. There were approximately 15 copies per chromosome for both BBR1-mRFP plasmids (Figure 4B). There was a significant difference in the plasmid copy number, depending on the selection marker, when *lacZ* was expressed with the pBBR1 ORI. There were almost eight copies of the plasmid per chromosome for gentamicin selection and less than one copy of the plasmid per chromosome for kanamycin selection. This low copy number was similar to the RSF1010-kan-mRFP cultures, which also produced very low *mrfp* expression (Supplementary Figure S6) and fluorescence. Unlike when kanamycin was used for selection, the RSF1010-gent-mRFP strain yielded nearly 40 copies of the plasmid per chromosome.

### Aerobic Versus Phototrophic/Anaerobic Gene Expression

*R. palustris* is photosynthetic in anaerobic conditions, providing the organism energy from light in addition to the energy it can obtain from organic compounds (Larimer et al., 2004). To take advantage of this important source of energy, synthetic biology tools for heterologous gene expression also need to be characterized during phototrophic growth. The most commonly used fluorescent reporters, developed from the jellyfish *Aequorea victoria*’s GFP, require oxygen to synthesize their chromophores (Drepper et al., 2007). In addition, the oxygen-independent flavin-binding fluorescent proteins are not bright enough to be clearly discernable above *R. palustris’* background fluorescence at their excitation and emission wavelengths (Mukherjee et al., 2013). Therefore, RNA and gDNA was extracted from the *R. palustris* BBR1-kan-mRFP strain grown aerobically and anaerobically (100 µE white light) for RT-qPCR (following the MIQE guidleines (Bustin et al., 2009)) and plasmid copy number tests (Materials and Methods). In addition, RNA was extracted from the BBR1-gent-mRFP, RSF1010-kan-mRFP, and RSF1010-gent-mRFP strains, and wild type *R. palustris* controls, all grown to mid-exponential phase.

**FIGURE 5:**
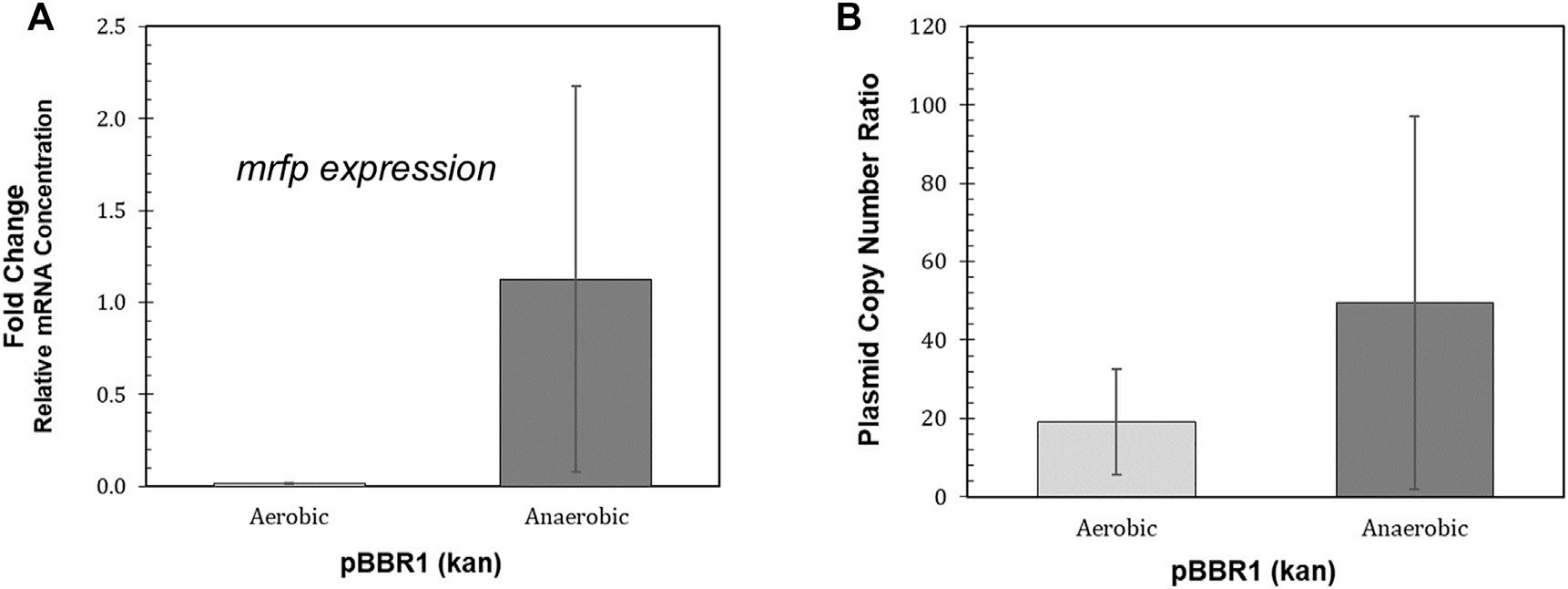
Performance of the *R. palustris* BBR1-kan-mRFP strain during phototrophic growth. **(A)** Relative mRNA concentration (*mrfp* relative to *16rRNA*) for the *R. palustris* BBR1-kan-mRFP strain when grown aerobically in the dark and anaerobically in the light (100 µE white light) (Materials and Methods). Two biological and two technical replicates were averaged for each strain. Error bars represent the population standard deviation. **(B)** Average plasmid copy number for the *R. palustris* BBR1-kan-mRFP strain grown aerobically in the dark and anaerobically in the light (100 µE white light) in duplicate. Copies of the kanamycin selection marker gene was compared to the single copy *dxs* gene in the chromosome by qPCR and an external standard curve (Materials and Methods). Error bars represent the population standard deviation.

Large variation in both *mrfp* expression relative to the reference gene and relative plasmid copy number was found for the biological replicates of the BBR1-kan-mRFP strain grown anaerobically (Figure 5). The average relative *mrfp* expression for the BBR1-kan cultures grown aerobically was minimal when compared to expression from the anaerobically grown cultures (Figure 5A). The aerobic relative *mrfp* expression from the BBR1-kan-mRFP strain was also compared to the other strains grown aerobically (BBR1-gent, RSF1010-kan and RSF1010-gent) (Supplementary Figure S4). The *mrfp* expression results of the aerobically grown strains was similar to the fluorescent fold change results (Figure 4A); the cultures for the two pBBR1 ORI strains had higher average relative *mrfp* expression than the RSF1010-gent culture, and the RSF1010-kan culture had no detectable *mrfp* expression. The plasmid copy number more than doubled to 50 copies per chromosome, when comparing the BBR1-kan-mRFP cultures grown anaerobically to those grown aerobically (Figure 5B).

### Genetic Context and Transcriptional Terminators

Transcription does not necessarily stop at the end of a gene, but can continue on to other genes on the same DNA strand (Chen et al., 2013; Kelly et al., 2019). The fluorescent reporter gene on the original BBR1 plasmids was preceded on the same DNA strand by the antibiotic resistance gene, which was not true for the same fluorescent reporter gene on the RSF1010 plasmids. Two transcriptional terminators, tonB and rrnC, were tested for their ability to reduce the transcriptional read-through from the antibiotic resistance gene, and both were compared to the strain with the *P*_*Lac*_ *-mrfp* expression cassette on the opposite strand of DNA in the BBR1-kan-plasmid (Materials and Methods). Both terminators significantly reduced transcriptional read-through in *E. coli*; although, the rrnC terminator was determined to be 25 times stronger than tonB (Chen et al., 2013).

**FIGURE 6:**
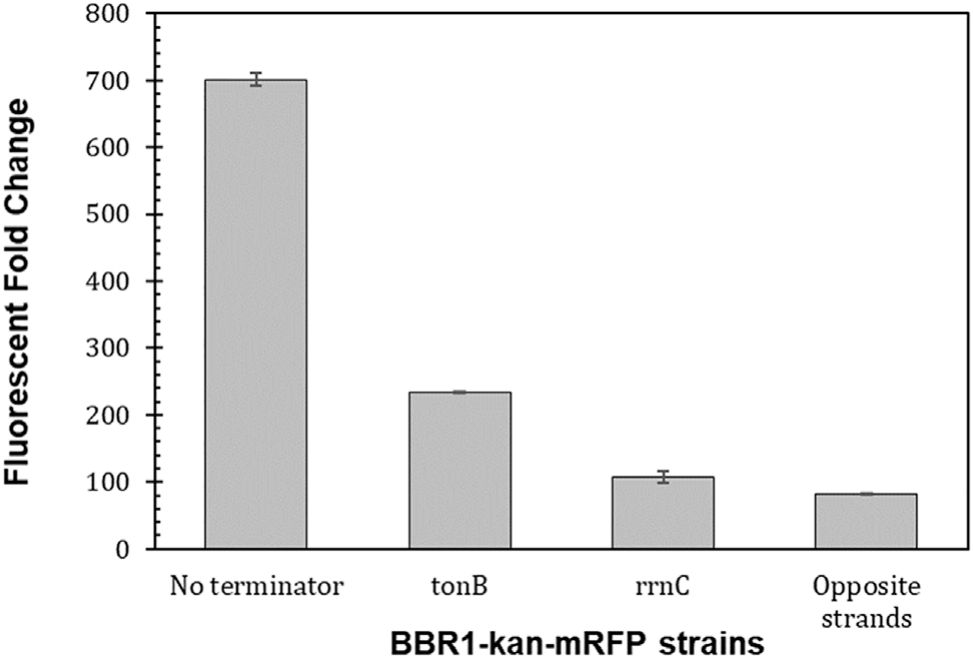
Transcriptional terminators reduce read-through from the preceding transcription unit. Fluorescent fold change average of *R. palustris* BBR1-kan-mRFP (no terminator), BBR1-kan-mRFP-tonB, BBR1-kan mRFP rrnC, and BBR1-kan-mRFP (opposite strand) strains grown in triplicate (Materials and Methods). Error bars represent the population standard deviation.

Moving the *P*_*Lac*_ *-mrfp* expression cassette to the opposite strand of DNA in the BBR1-kan-mRFP plasmid, reduced the fluorescent fold change more than eight times to 82 (Figure 6), which is similar to the fluorescent fold change from the RSF1010-gent-mRFP plasmid, at 87 (Figure 4A). Inserting the tonB terminator between *kan* and *mrfp* resulted in a three-fold decrease in fluorescent fold change in the *R. palustris* strain while the rrnC terminator produced more than a six-fold reduction.

### Predicting Changes in Protein Production Based on the 5’ UTR

**FIGURE 7:**
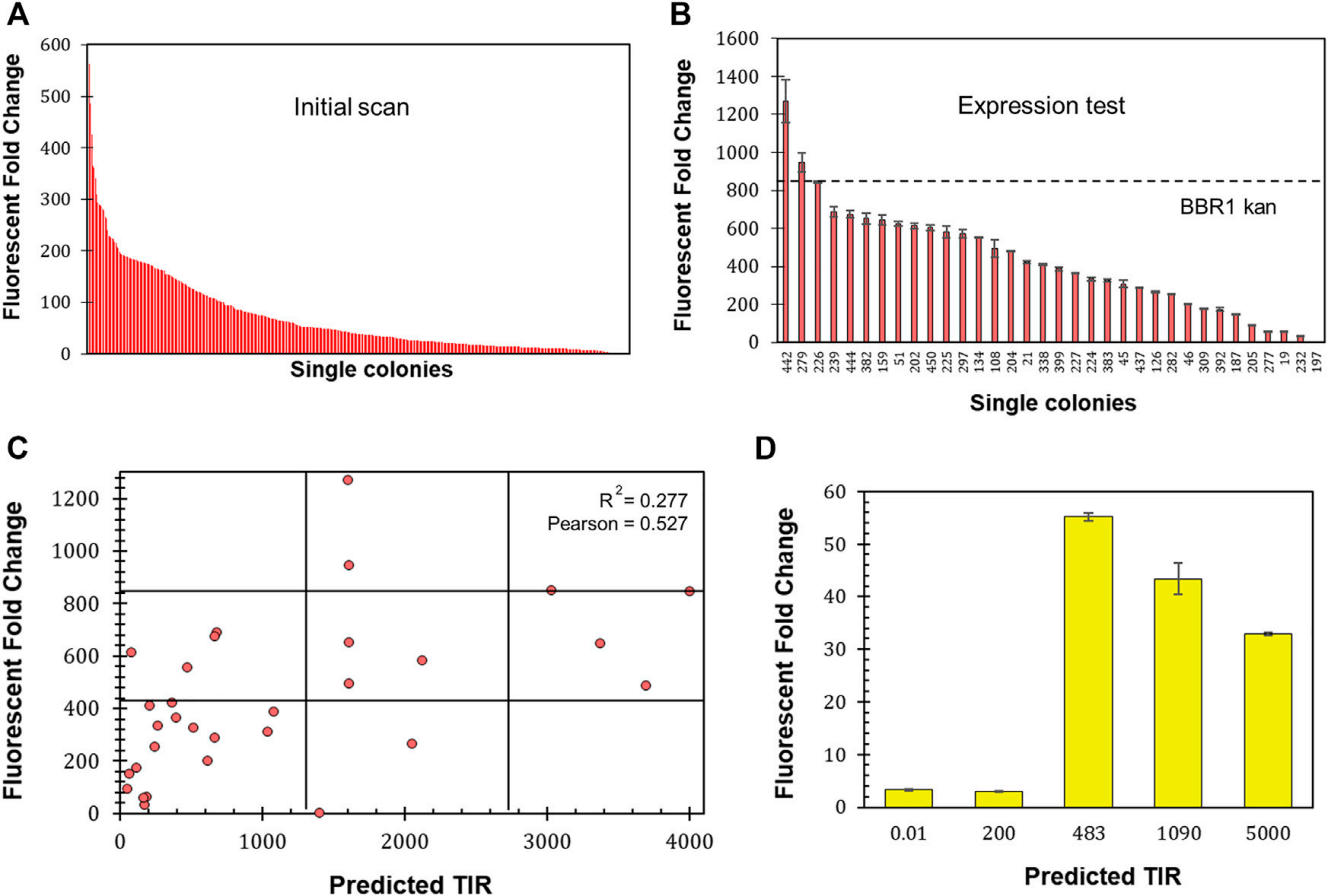
Determining 5′ UTR design guidelines for *R. palustris*. **(A)** Fluorescent fold change scan of *R. palustris* BBR1-kan-mRFP (RBS library) single colonies (Materials and Methods). **(B)** Average fluorescent fold change of select *R. palustris* BBR1-kan-mRFP (RBS library) strains grown in triplicate (Materials and Methods). Error bars represent the population standard deviation. **(C)** Comparison of the predicted translation initiation rate (TIR) versus the fluorescent fold change average of the select *R. palustris* BBR1-kan (RBS library)-mRFP strains. The grid lines divide each axis in thirds to aid in analysis. **(D)** Average fluorescent fold change of *R. palustris* BBR1-kan (0.01, 200, 483, 1,090, and 5,000)-5′ UTR-eYFP strains grown in triplicate (Materials and Methods). Error bars represent the population standard deviation.

The 5′ untranslated region (UTR) of mRNA significantly impacts protein production through its participation in transcription and the stability of the transcript, as well as through translation (Le et al., 2020). To investigate the range of expression that could be achieved and whether the level of fluorescent protein production could be predicted, the six bases of *mrfp*’s ribosome site (RBS) were randomized on the BBR1-kan-mRFP plasmid (Materials and Methods). A scan of 450 colonies yielded an expression range of 562-fold (Figure 7A). 32 colonies from across the range of normalized fluorescence, plus the original BBR1-kan-mRFP strain, were then tested per the Fluorescent Fold Change Measurement protocol, which produced nearly a 1,300-fold range of expression (Figure 7B). The sequence of the RBS was determined for the 32 colonies and used to generate a predicted translation initiation rate (TIR) with the DeNovo DNA RBS calculator (Salis et al., 2009; Borujeni et al., 2014;Borujeni and Salis, 2016; Borujeni et al., 2017; Reis and Salis, 2020; Cetnar and Salis, 2021). The predicted TIR versus the fluorescent fold change was then plotted for each strain (Figure 7C). The coefficient of determination (*R* ^2^) was less than 0.3, suggesting a significant variation in fluorescence based on the TIR. The Pearson correlation coefficient was greater than 0.5, which does imply a moderate strength of association.

The optimize expression levels function of the DeNovo DNA RBS calculator was then used to design 5′ UTRs with higher (1,090 and 5,000) and lower (0.02 and 200) TIRs than the original BBR1-kan-eYFP plasmid, TIR of 483. The entire 5’ UTR of *eyfp* in the BBR1-kan-eYFP plasmid was then replaced with sequences designed by the calculator. The fluorescent fold change was determined for the four subsequent strains following the same protocols used previously (Materials and Methods). The fluorescent fold change for the strains with high TIRs was lower than the original strain, 33-fold and 43-fold versus the original 55-fold (Figure 7D). The strains with the low TIRs produced very low fluorescent fold change, both approximately 3-fold.

### Harnessing pRPA for Heterologous Protein Production

**FIGURE 8:**
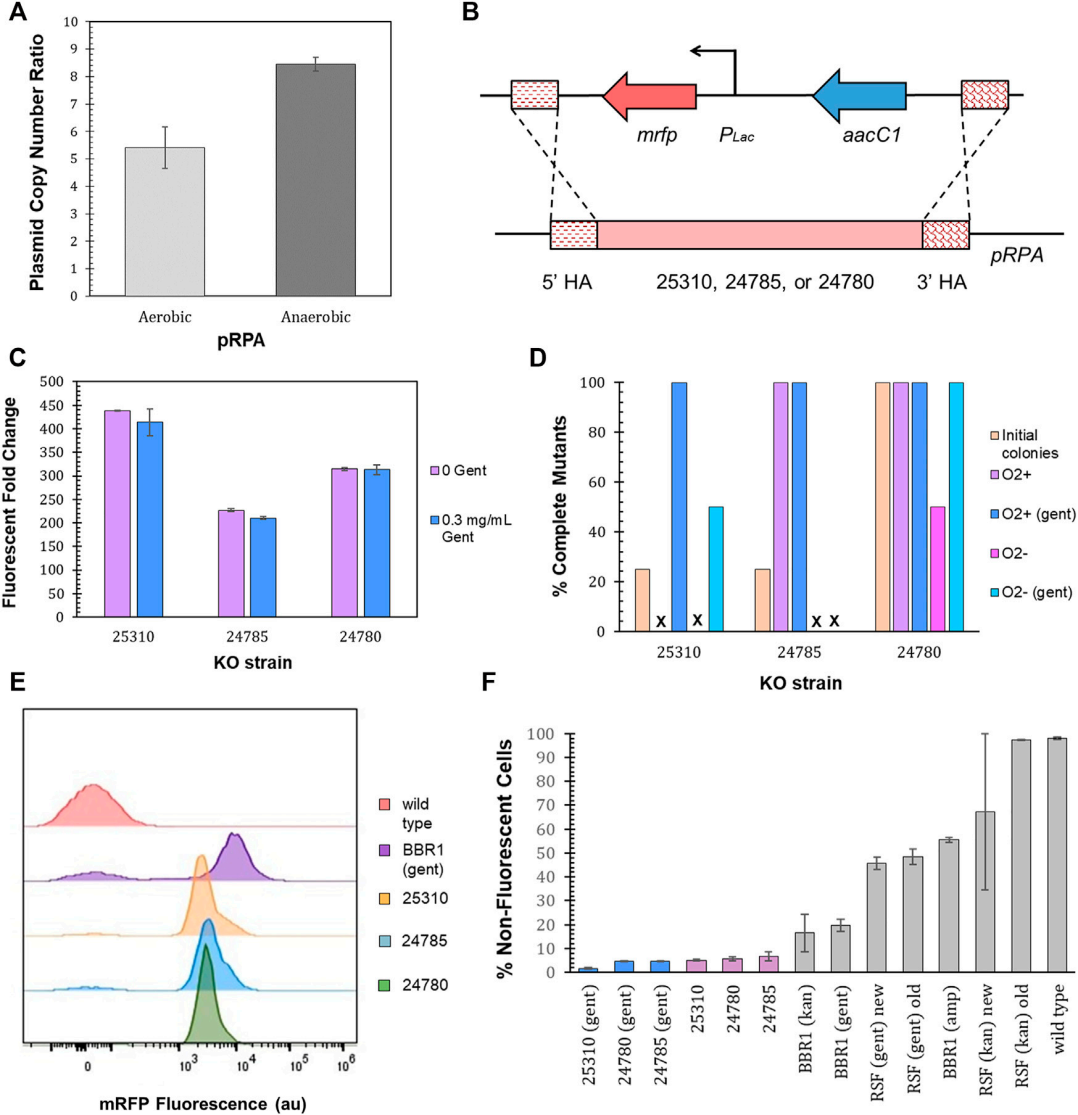
pRPA as a heterologous gene expression vector. **(A)** Average pRPA copy number for wild type *R. palustris* grown aerobically in the dark and anaerobically in the light (100 µE white light) in duplicate. Copies of the plasmid’s replication protein gene (TX73_RS24750) was compared to the single copy *dxs* gene in the chromosome by qPCR and an external standard curve (Materials and Methods). Error bars represent the population standard deviation. **(B)** Schematic of the *P*_*Lac*_ *-mrfp* expression cassette and a gentamicin selection marker integrated into one of three locations, TX73_RS25310, TX73_RS24785, TX73_RS24780, in *R. palustris*’ endogenous plasmid, pRPA, by double homologous recombination. **(C)** Fluorescent fold change average of *R. palustris* pRPA-25310-mRFP-Gent, pRPA-24785-mRFP-Gent, and pRPA-24780-Gent strains grown in triplicate either without any antibiotic or with 0.3 mg/ml gentamicin sulfate. The data is representative of the first test with new cultures as well as the test after three rounds of dilution and regrowth (Materials and Methods). Error bars represent the population standard deviation. **(D)** Comparison of the percent complete mutants (those that do not produce an amplicon from PCR with primers binding to the DNA that is removed and also produce the correct sequence from PCR with primers outside of the homology arms) for the three pRPA locations. **(E)** Representative flow cytometry results of mRFP fluorescence from *R. palustris* wild type, BBR1-gent-mRFP, pRPA-25310-mRFP-Gent, pRPA-24785-mRFP-Gent, and pRPA-24780-Gent strains grown in triplicate. The growth procedure, media, and antibiotics are the same as for the fluorescent fold change test in Figure 8C (Materials and Methods). **(F)** Average percent non-fluorescent cells for *R. palustris* strains grown in triplicate as determined by flow cytometry (Materials and Methods). Error bars represent the population standard deviation.

*R. palustris*’ active partitioning system ensures its endogenous plasmid, pRPA, is segregated into daughter cells (Debaugny et al., 2018), unlike non-native plasmids (Meyer, 2009). Employing *R. palustris*’ endogenous plasmid could therefore address the problem of plasmid loss. pRPA encodes nine potential genes (Larimer et al., 2004) with five of the nine open reading frames annotated. Three of the open reading frames for hypothetical proteins are clustered together and one sits between genes for a replication protein and a protein associated with allocating the chromosomes and plasmids during cell division (Ebersbach and Gerdes, 2005). The copy number of pRPA was determined to average more than five copies per chromosome for aerobic cultures and more than eight for anaerobic cultures (100 µE white light) (Figure 8A) using qPCR and *R. palustris*’ gDNA as discussed earlier (Materials and Methods). In addition, there was minimal variation in the copy number for anaerobic cultures in contrast to the BBR1-kan-mRFP plasmid (Figure 5B).

Double homologous recombination with a p15A suicide plasmid (Materials and Methods) was then utilized to replace each of the three open reading frames (the function of which are currently unknown) that are clustered together (TX73_RS25310, TX73_RS24785, or TX73_RS24780) with a gentamicin selection marker and the *P*_*Lac*_ *-mrfp* expression cassette, coded on the same DNA strand without a terminator between them (Figure 8B). Inserting the expression cassette into a non-coding region of the plasmid was initially considered, yet the only sizeable non-coding region (∼500 bp) was directly adjacent to the gene encoding the plasmid’s replication protein. Since the regulatory regions of this gene are unknown, there were concerns about affecting the plasmid’s replication. As a result, this option was not pursued. After transformation with the suicide plasmids, four colonies were selected for each open reading frame replacement. The loss of the suicide plasmid and the incorporation of the expression cassette and selection marker was determined by colony PCR and sequencing of the PCR products. None of the colonies still harbored the suicide plasmid. One of four colonies for TX73_RS25310 and TX73_RS24785 (25310 and 24785) had completely integrated the expression cassette and selection marker into all copies of pRPA (Figure 8D), as determined by the absence of a PCR product for primers that only bound to the DNA being replaced as well as an amplicon of the expected size and correct sequence for primers that bound outside of the homology arms. All four colonies for 24780 also showed complete segregation. The fluorescent fold change measurement protocol was then followed for cultures grown from a single colony that demonstrated complete segregation for each open reading frame replacement (Materials and Methods). The 25310 strain produced the highest fluorescent fold change, 400, while the fluorescent fold change for the 24785 and 24780 strains was 200 and 300 respectively (Figure 8C).

The homogeneity of cultures that had shown complete segregation, as determined by the absence of a PCR product of the expected size for primers that only bound to the DNA being replaced, was investigated over time by diluting the cultures just tested into media without gentamicin and also diluting the same culture into media with gentamicin, letting them reach stationary phase for at least 24 h, and repeating the dilution and growth two more times. The presence or absence of gentamicin was maintained when diluting the last two rounds. Three rounds of dilution and growth were completed for cultures grown aerobically and one round was completed for cultures grown anaerobically (100 µE white light). Colony PCR, sequencing, and the fluorescence test was repeated at the end of the third aerobic round and the end of the first anaerobic round. There was no change in the fluorescent fold change, tested only in aerobic cultures (data not shown). The homogeneity of the cultures was dependent on the growth conditions, the presence of gentamicin in the media, and the open reading frame (Figure 8D). For all cultures grown aerobically with gentamicin, there was no appearance of wild type cells after the third round of dilutions, as determined by the absence of a PCR product of the expected size for primers that only bound to the DNA being replaced. For cultures grown anaerobically with gentamicin, only strain 24780 did not show the addition of wild type cells for all colonies tested. A negative (no DNA) and positive control (lysed wild type) were included in every PCR reaction to reduce the probability of errant reactions.

Flow cytometry was then employed to further investigate the homogeneity of the cultures for all three strains, 25310, 24785, and 24780. Triplicates of each strain were grown with gentamicin in addition to triplicates of each strain without gentamicin, all in aerobic conditions for the flow cytometry test. Figure 8E is representative of the results. The averages and standard deviations are presented in Figure 8F. Complete flow cytometry results for these strains can be found in Supplementary Figures S17–22. There was little variation in the number of cells with just the background fluorescence for the three strains utilizing pRPA, regardless of gentamicin in the media. The cultures of strain 24785 grown without gentamicin saw the largest average number of cells, 6.7%, with just the background fluorescence of the three strains and two growth conditions. The variation was also highest for strain 24785 grown without gentamicin, ±1.8%. This worst case pRPA mutant had a statistically lower number of cells with just the background fluorescence as compared to the most similar non-native plasmid BBR1-gent-mRFP (student’s two-tail *t*-test, *p* < 0.05), which requires gentamicin for the plasmid to be maintained.

## Discussion

*R. palustris*’ multiple modes of metabolism endow it with many valuable biochemical capabilities including utilizing energy from sunlight, fixing carbon, catabolizing recalcitrant aromatic compounds, and fixing nitrogen/producing hydrogen (Larimer et al., 2004). Furthermore, *R. palustris* can remain metabolically active in a non-growing state for months when supplied with just light and organic carbon (Gosse et al., 2010). Even with all of these beneficial traits, published work for engineering this microbe has been limited. *R. palustris*’ intrinsic resistance to antibiotics (Larimer et al., 2004) and the lack of basic synthetic biology tools that have been characterized for this PNSB are impediments to harnessing its potential. To address these problems, this work has focused on establishing a baseline understanding of the behavior of genetic parts (including selection markers, origins of replication, fluorescent reporters, terminators, and 5’ untranslated regions) that are fundamental to future engineering of this remarkable microorganism. Furthermore, this new knowledge provided the tools needed to take a significant step forward in establishing predictable heterologous gene expression in the chassis by employing its endogenous plasmid.

### Selection Pressure

*R. palustris*’ antibiotic resistance makes choosing a selection marker for gene expression in this bacterium critical. While the microbe was most sensitive to ampicillin in aerobic conditions, only 50% of the cells in the cultures were fluorescent at 50 μg/ml, the concentration that led to the highest fluorescence from strains harboring the BBR1-amp-mRFP plasmid. In addition, *R. palustris* was not sensitive to ampicillin in anaerobic growth conditions with 100 µE white light. The gene for a chloramphenicol acetyltransferase (CAT) is annotated in *R. palustris*’ genome and was expressed in both aerobic and anaerobic (100 µE white light) conditions. CAT attaches an acetyl group to the antibiotic, which prevents chloramphenicol from binding to the bacterium’s ribosome (Shaw, 1983). Removing *cat* did not increase *R. palustris*’ sensitivity to chloramphenicol, pointing to another mechanism that is responsible for the bacterium’s resistance.

Kanamycin is frequently used for selection in *R. palustris* (Brown et al., 2020; Doud et al., 2017; Heiniger et al., 2015; Hirakawa et al., 2012). The BBR1-kan-mRFP strain produced strong mRFP fluorescence, *mrfp* expression, and plasmid copy number while the RSF1010-kan-mRFP strain did not for any metric. Swapping the kanamycin selection marker with a gentamicin selection marker resulted in *R. palustris* strains that produced similar strong mRFP fluorescence, *mrfp* expression, and plasmid copy number from the BBR1-mRFP plasmid. In addition, the BBR1-gent-LacZ strain demonstrated strong β-galactosidase activity and a relative plasmid copy number similar to pRPA. For the BBR1-kan-LacZ strain, the β-galactosidase activity was nearly three-fold less than the strain with gentamicin selection and the plasmid copy number was less than one per chromosome. mRFP fluorescence, *mrfp* expression, and plasmid copy number were also maintained in the RSF1010-gent-mRFP strain. This suggests that gentamicin produces better selection pressure than kanamycin in *R. palustris.* This might be related to gentamicin stopping growth of the wild type strain in 13 h as opposed to more than 30 h required for kanamycin. In addition, wild type cultures grown anaerobically with gentamicin did not recover after removing the antibiotic while wild type cultures grown anaerobically with kanamycin produced multiple single colonies after allowing the culture to recover without the antibiotic.

When testing *R. palustris*’ sensitivity to antibiotics, spectinomycin allowed for even less growth than gentamicin, but over a significantly longer time. Spectinomycin’s resistance gene was therefore not tested for selection for this work, but it is a possibility in the future. Tetracycline was not even considered because of its light sensitivity, but it might be useful for selection in just aerobic conditions.

*R. palustris*’ intrinsic resistance to antibiotics has been attributed to its 22 unique resistance-nodulation-cell division (RND) pumps, which is more than has been discovered in any other bacterium (Larimer et al., 2004). RND pumps shuttle the substrate across the membrane and into the external medium, minimizing the internal substrate concentration. The energy source for RND pumps is a proton gradient (Fernando and Kumar, 2013), which can be maintained after growth stops due to *R. palustris*’ cyclic photophosphorylation. This could help explain the increased variation in the BBR1 and the pRPA knockout strains in anaerobic/photosynthetic conditions. These efflux pumps often have broad substrate specificity (Fernando and Kumar, 2013); in other words, one RND pump may be able to reduce the internal concentration of multiple antibiotics. Generally, these pumps are tightly regulated by interactions between local repressors and global regulators. As specific mechanisms of regulation are determined, possible solutions to the antibiotic resistance of a potential synthetic biology chassis could involve engineering the regulators to be less sensitive to the antibiotic(s) used for selection or altering the balance between the repressors and the inducers of the pumps.

Since there has been some success using kanamycin and gentamicin selection markers, higher concentrations of the antibiotics could also be tested to see if selection pressure increases. More effective selection pressure might increase the plasmid copy number for the RSF1010-kan-mRFP and BBR1-kan-LacZ strains to more than one per chromosome and decrease the number of non-fluorescent cells for the RSF1010-gent-mRFP cells to less than 50% of the culture. Higher antibiotic concentrations started to affect growth of *R. palustris* mutants, with a 17% decrease in growth at 600 μg/ml kanamycin or gentamicin as compared to wild type and a 26% decrease at 1,200 μg/ml. Therefore, any increase in plasmid copy number or decrease in non-fluorescent cells would have to be weighed against weaker growth.

Auxotrophies could also be investigated to identify a selection mechanism that does not involve antibiotics (Seif et al., 2020). Since *R.* palustris’ genome does not have an annotated pathway for *p-*aminobenzoic acid, it is commonly added to its minimal media (Allen et al., 2015; Kim and Harwood, 1991). Yet, *R. palustris* grew equivalently with and without *p*-aminobenzoic acid in its media when sodium acetate was the carbon source, in aerobic and anaerobic (100 µE white light) conditions. As there are no other auxotrophies suggested by the bacterium’s genome, one would need to be generated to create the selection pressure.

### Origins of Replication and Plasmid Maintenance

pBBR1 and RSF1010 are among the many origins of replication that have been identified as having a broad host range (Jain and Srivastava, 2013). While pBBR1 has been used for gene expression in *R. palustris* previously (Braatsch et al., 2006; Huang et al., 2010; Heiniger and Harwood, 2015), its maintenance and copy number had not been investigated in the microbe. RSF1010 belongs to a different incompatibility group than pBBR1 (Del Solar et al., 1998; Meng et al., 2013). The RSF1010 ORI uses the strand displacement mechanism and belongs to the IncQ group, which requires three plasmid-encoded proteins to initiate replication (Del Solar et al., 1998). The pBBR1 ORI does not belong to the IncP, IncQ, or the IncW groups and replicates similar to the rolling circle mechanism used by small plasmids in Gram-positive bacteria (Antoine & Locht, 1992). RSF1010s stability and copy number in *R. palustris* were investigated since this would open the possibility of using the two heterologous plasmids together in the future. *R. palustris’* native plasmid’s (pRPA’s) replication mechanism has not been determined and its compatibility with plasmids commonly used in synthetic biology has not been investigated. This leaves the possibility that competition for replication machinery could exist between it and pBBR1. pRPA’s single replication initiator protein, RepA, is encoded on the plasmid, but it shows no amino acid similarity (per Protein Blast) to the single replication protein encoded on BBR1, reducing, but not eliminating, the possibility of incompatibility.

The origin of replication and selection marker both influence plasmid maintenance, making it difficult to tease apart precise rules for use in this microbe. Both ORIs produced heterogeneous populations as shown by flow cytometry. The current data does not validate that the non-fluorescent cells lost their plasmid. Yet, the absence of mutations in *mrfp* for all cultures (as determined by sequencing of PCR products from colony PCR), as well as a plasmid copy number of less than one per chromosome, the lack of *mrfp* expression (relative RNA concentration), and the absence of fluorescence for the majority of the cells for the RSF1010-kan-mRFP strain does suggest the absence of the plasmid in some cases. Cell sorting could be used in the future to help answer this question more definitively. If the non-fluorescent cells for the RSF1010-gent-mRFP strain lost their plasmid, our data suggests that it is not due to low plasmid copy number. This strain’s average plasmid copy was higher than that from the BBR1-gent-mRFP strain. While it is difficult to tease apart the contributions of selection pressure and plasmid replication, the two strains with the lowest plasmid copy numbers both relied on kanamycin selection, suggesting a significant role for selection pressure in these cases.

### Phototrophy and Tool Performance

To take advantage of the energy produced from *R. palustris*’ phototrophic (anaerobic) metabolism, the synthetic biology tools used to engineer the microbe also need to be characterized during this mode of growth. Due to the oxygen requirement of most fluorescent reporters and the bacterium’s background fluorescence, easy characterization through the use of a fluorescent reporter is not feasible. *mrfp* expression and plasmid copy number, both determined by qPCR, produced larger variation than what was seen from the same tests during aerobic growth. The source of this variation has not been determined.

*R. palustris’* use of cyclic photophosphorylation during anaerobic/photosynthetic growth enables the maintenance of a proton gradient after cultures stop growing (McKinlay, 2014). This could allow RND pumps to maintain a lower internal antibiotic concentration continually in anaerobic conditions while the same would not be true in aerobic conditions after growth stops. Additionally, *R. palustris*’ photoheterotrophic metabolism is sensitive to how much light the microbe can absorb (Alsiyabi et al., 2019; Navid et al., 2019). Differences in light intensity are somewhat common throughout the growth chamber, which could lead to differences in the level of photosynthesis between the cultures in the chamber and potentially significant variations in gene expression and relative plasmid copy number between culture tubes. The constitutive promoter *P*_*Lac*_ (used to express all reporters in this work) has not been characterized in *R. palustris;* although, it has been used previously in complementation experiments in *R. palustris* (Huang et al., 2010; Allen et al., 2015) to restore the functionality of the knocked-out gene. There is a possibility that the variation in gene expression is related to the promoter’s ability to recruit RNA polymerase in anaerobic conditions consistently.

Even with this variation, the results for the BBR1-kan-mRFP strain in anaerobic conditions were hopeful. The relative mRNA concentration and plasmid copy numbers of all BBR1-kan-mRFP replicates grown anaerobically were still equivalent or higher than the same metric used for the respective aerobic cultures.

### Transcriptional Terminators

Terminators increase predictable gene expression by insulating the gene of interest from other nearby transcription units (Chen et al., 2013; Kelly et al., 2019), making them an important tool for regulating heterologous gene expression. Studies of terminator performance in *R. palustris* were not found. Furthermore, the construction of the BBR1-kan-mRFP (opposite strand) strain indicated that the original plasmid did not have an effective terminator between the selection marker and the expression cassette, *P*_*Lac*_ *-mrfp*. Therefore, two terminators that had performed significantly different in *E. coli*, rrnC was 25 times stronger than tonB, were selected (Chen et al., 2013). Both terminators worked well on the BBR1-kan-mRFP plasmid in *R. palustris,* suggesting that other terminators characterized in *E. coli* could work well too.

### Designing 5’ UTRs

Randomizing the six base pair ribosome binding site (RBS) for *mrfp* produced a wide range of expression levels. The question was whether or not the expression level could be predicted before the 5′ untranslated region (UTR) was changed. Choosing the expression level is particularly important for balancing metabolic pathways and producing enzymes whose products are toxic at high concentrations. Comparing the translation initiation rates predicted by the RBS calculator to the normalized fluorescence for sequences across the range of expression revealed a moderate level of association, but poor predictability. In general, TIRs predicted to produce low expression generally produced low fluorescence while high TIRs all underperformed. The 5’ UTRs designed for *eyfp* fit into the same pattern. Therefore, the usefulness of this tool depends on how it is applied. It does not work well for creating a *R. palustris* strain with specific level of expression, but it could be used to create a handful of strains that would then be tested, potentially reducing the number of tests needed before a strain with the desired expression level is found.

### Harnessing pRPA for Heterologous Protein Production

Employing *R. palustris*’ endogenous plasmid could be an answer to the problems that occurred in the BBR1-kan-mRFP strain, instability of protein production with a plasmid copy number less than one per chromosome. Furthermore, if integration of the gene(s) of interest into all copies of pRPA is achieved, selection pressure should not be needed, which is preferred for biotechnology applications. In addition, the plasmid copy number for pRPA showed little variation in aerobic and anaerobic conditions, which would improve the predictability of gene expression. pRPA’s plasmid copy numbers were also statistically similar to the plasmid copy numbers for the BBR1 plasmids from cultures grown aerobically (student’s two-tail *t*-test, *p* > 0.05). This indicates that pRPA should be a good alternative to non-native plasmids for heterologous gene expression.

During aerobic growth, more than 90% of the cells in the pRPA cultures for all three open reading frames were fluorescent, as compared to 80% for the BBR1-gent-mRFP strain. The level of normalized fluorescence was dependent on the open reading frame that was replaced, but not the presence of gentamicin in the media, even across the span of three rounds of dilution and regrowth. Gentamicin did ensure the absence of wild type cells when the all three strains were grown aerobically, but was less successful when strain 24785 and 25310 were grown anaerobically. Interestingly, the *R. palustris* Δ*cat* strain constructed through sucrose counterselection also produced PCR products that indicated the presence of wild type cells when a double knockout was attempted. Any wild type cells would probably have a faster growth rate, at least in some conditions, than the cells producing heterologous proteins and could therefore eventually outcompete the mutants. This makes the development of strong selection pressure a priority.

Stable and predictable heterologous protein production can be achieved in the metabolically robust *R. palustris* when the genetic parts have been well characterized for the condition(s) of interest and their limitations are understood. It was not intuitive for the pink bacterium’s background fluorescence to be lowest for mRFP’s excitation and emission wavelengths. The two transcriptional terminators tested did a good job of insulating the expression cassette. Both of the BBR1 and RSF1010 plasmids were stably maintained over time with gentamicin selection for *mrfp* and *lacZ* expression. The predicted TIR was moderately associated with the measured fluorescence, which could be used to reduce the number of strains built before one with the desired level of expression is found. Furthermore, gene expression, heterologous plasmid copy number, and the homogeneity of cultures with complete segregation over time was more consistent for aerobic conditions than for anaerobic conditions; yet, there is a potential for high levels of protein production when the bacterium is performing photosynthesis. Finally, utilizing pRPA as an expression vector shows promise. Significant levels of mRFP were generated from the endogenous plasmid, and two of the integration sites did not require selection pressure in aerobic conditions after strain validation to remain free of wild type cells. While not all of *R. palustris*’ secrets have been unlocked, this work has begun to clarify rules for expressing heterologous genes in the complex microbe, laying the groundwork for future engineering endeavors that harness its unique biochemical potential.

## Data Availability

The original contributions presented in the study are included in the article/Supplementary Material, further inquiries can be directed to the corresponding author.
