# Supplementary material for: Synthetic Biology Tool Development Advances Predictable Gene Expression in the Metabolically Versatile Soil Bacterium Rhodopseudomonas palustris
Source: Front Bioeng Biotechnol. 2022 Mar 16;10:800734. doi: 10.3389/fbioe.2022.800734 (PMC8966681; doi:10.3389/fbioe.2022.800734)
Supplement: Supplementary file 1 [file DataSheet1.docx]

**SUPPLEMENTAL MATERIAL**

**Synthetic Biology Tool Development Advances Predictable Gene Expression in the Metabolically Versatile Soil Bacterium *Rhodopseudomonas palustris***

Cheryl Immethun, Mark Kathol, Taity Changa, and Rajib Saha

**Supplementary Table I.** Plasmids/Strains used in this work.

| **Name** | **Parts** | **Type and Source** |
| --- | --- | --- |
| pJQ200SK | p15A ori; *gent^R^; P_sacb_:sacb* | Integrative  (Quant, Hynes, 1993) |
| pBBR1MCS-2 | pBBR1 replicon; *kan^R^*; *P_Lac_* | Replicative  (Kovach et al., 1995) |
| pBbB7a-GFP | *P_amp:_amp^R^; gfpuv* | Replicative  (Lee et al., 2011) |
| pSL3068 | RSF1010 replicon; *eyfp* | Replicative  (Knoot et al., 2019) |
| pCMrfp | *mrfp* | Replicative  (Bi et al., 2013) |
| pRPA | *R. palustris* endogenous plasmid | Replicative |
| pΔcat | p15A ori; *5’HA CAT; 3’HA CAT; gent^R^; P_sacb_:sacb* | Integrative  This Study |
| BBR1-kan-GFPuv | pBBR1 replicon; *kan^R^; P_Lac_:gfpuv* | Replicative  This Study |
| BBR1-kan-483-eYFP | pBBR1 replicon; *kan^R^; P_Lac_:eyfp* | Replicative  This Study |
| BBR1-kan-mRFP | pBBR1 replicon; *kan^R^; P_Lac_:mrfp* | Replicative  This Study |
| BBR1-kan-LacZ | pBBR1 replicon; *kan^R^; P_Lac_:lacZ* | Replicative  This Study |
| BBR1-kan-mRFP (opposite strand) | pBBR1 replicon; *kan^R^; P_Lac_:mrfp* | Replicative  This Study |
| BBR1-amp-mRFP | pBBR1 replicon; *P_amp_:amp^R^; P_Lac_:mrfp* | Replicative  This Study |
| BBR1-gent-mRFP | pBBR1 replicon; *gent^R^; P_Lac_:mrfp* | Replicative  This Study |
| BBR1-gent-LacZ | pBBR1 replicon; *gent^R^; P_Lac_:lacZ* | Replicative  This Study |
| BBR1-kan-mRFP-tonb | pBBR1 replicon; *kan^R^; ton B; P_Lac_:mrfp* | Replicative  This Study |
| BBR1-kan-mRFP-rrnc | pBBR1 replicon; *kan^R^; rrnc; P_Lac_:mrfp* | Replicative  This Study |
| BBR1-kan-0.01-eYFP | pBBR1 replicon; *kan^R^; P_Lac_:0.01 5’ UTR eyfp* | Replicative  This Study |
| BBR1-kan-200-eYFP | pBBR replicon; *kan^R^; P_Lac_:200 5’ UTR eyfp* | Replicative  This Study |
| BBR1-kan-1090-eYFP | pBBR replicon; *kan^R^; P_Lac_:1090 5’ UTR eyfp* | Replicative  This Study |
| BBR1 kan 5000 eYFP | pBBR1 replicon; *kan^R^; P_Lac_:5000 5’ UTR eyfp* | Replicative  This Study |
| pRPA-24780-mRFP-Gent | P15A ori; *3’HA 24780; gent^R^; P_Lac_:mrfp; 5’HA 24780* | Integrative  This Study |
| pRPA-24785-mRFP-Gent | p15A ori; *3’HA 24785; gent^R^; P_Lac_:mrfp; 5’HA 24785* | Integrative  This Study |
| pRPA-25310-mRFP-Gent | p15A ori; *3’HA 25310; gent^R^; P_Lac_:mrfp; 5’HA 25310* | Integrative  This Study |

**Supplementary Table II.**  List of genetic parts used in this work.

| Part name | Type and source | DNA sequence |
| --- | --- | --- |
| *amp^R^* | Antibiotic resistance gene  (Lee et al., 2011) | atgagtattcaacatttccgtgtcgcccttattcccttttttgcggcattttgccttcctgtttttgctcacccagaaacgctggtgaaagtaaaagatgctgaagatcagttgggtgcacgagtgggttacatcgaactggatctcaacagcggtaagatccttgagagttttcgccccgaagaacgttttccaatgatgagcacttttaaagttctgctatgtggcgcggtattatcccgtattgacgccgggcaagagcaactcggtcgccgcatacactattctcagaatgacttggttgagtactcaccagtcacagaaaagcatcttacggatggcatgacagtaagagaattatgcagtgctgccataaccatgagtgataacactgcggccaacttacttctgacaacgatcggaggaccgaaggagctaaccgcttttttgcacaacatgggggatcatgtaactcgccttgatcgttgggaaccggagctgaatgaagccataccaaacgacgagcgtgacaccacgatgcctgtagcaatggcaacaacgttgcgcaaactattaactggcgaactacttactctagcttcccggcaacaattaatagactggatggaggcggataaagttgcaggaccacttctgcgctcggcccttccggctggctggtttattgctgataaatctggagccggtgagcgtgggtctcgcggtatcattgcagcactggggccagatggtaagccctcccgtatcgtagttatctacacgacggggagtcaggcaactatggatgaacgaaatagacagatcgctgagataggtgcctcactgattaagcattggtaa |
| *kan^R^* | Antibiotic resistance gene  (Kovach et al., 1995) | atgattgaacaagatggattgcacgcaggttctccggccgcttgggtggagaggctattcggctatgactgggcacaacagacaatcggctgctctgatgccgccgtgttccggctgtcagcgcaggggcgcccggttctttttgtcaagaccgacctgtccggtgccctgaatgaactgcaggacgaggcagcgcggctatcgtggctggccacgacgggcgttccttgcgcagctgtgctcgacgttgtcactgaagcgggaagggactggctgctattgggcgaagtgccggggcaggatctcctgtcatctcaccttgctcctgccgagaaagtatccatcatggctgatgcaatgcggcggctgcatacgcttgatccggctacctgcccattcgaccaccaagcgaaacatcgcatcgagcgagcacgtactcggatggaagccggtcttgtcgatcaggatgatctggacgaagagcatcaggggctcgcgccagccgaactgttcgccaggctcaaggcgcgcatgcccgacggcgaggatctcgtcgtgacccatggcgatgcctgcttgccgaatatcatggtggaaaatggccgcttttctggattcatcgactgtggccggctgggtgtggcggaccgctatcaggacatagcgttggctacccgtgatattgctgaagagcttggcggcgaatgggctgaccgcttcctcgtgctttacggtatcgccgctcccgattcgcagcgcatcgccttctatcgccttcttgacgagttcttctga |
| *gent^R^* | Antibiotic resistance gene  (Quandt and Hynes, 1993) | atgttacgcagcagcaacgatgttacgcagcagggcagtcgccctaaaacaaagttaggtggctcaagtatgggcatcattcgcacatgtaggctcggccctgaccaagtcaaatccatgcgggctgctcttgatcttttcggtCgtgagttcggagacgtagccacctactcccaacatcagccggactccgattacctcgggaacttgctccgtagtaagacattcatcgcgcttgctgccttcgaccaagaagcggttgttggcgctctcgcggcttacgttctgcccaagtttgagcagccgcgtagtgagatctatatctatgatctcgcagtctccggcgagcaccggaggcagggcattgccaccgcgctcatcaatctcctcaagcatgaggccaacgcgcttggtgcttatgtgatctacgtgcaagcagattacggtgacgatcccgcagtggctctctatacaaagttgggcatacgggaagaagtgatgcactttgatatcgacccaagtaccgccacctaa |
| *tonB* | Terminator  (Postle and Good, 1983) | agtcaaaagcctccggtcggaggcttttgact |
| *rrnC* | Terminator  (Young, 1979) | cagataaaaaaaatccttagctttcgctaaggatgatttct |
| *P_Lac_* | Promoter  (Kovach et al., 1995) | ggcagtgagcgcaacgcaattaatgtgagttagctcactcattaggcaccccaggctttacactttatgcttccggctcgtatgttgtgtggaattgtgagcggataacaat |
| *P_SacB_* | Promoter  (Quant, Hynes, 1993) | cacatatacctgccgttcactattatttagtgaaatgagatattatgatattttctgaattgtgattaaaaaggcaactttatgcccatgcaacagaaactataaaaaatacagagaatgaaaagaaacagatagattttttagttctttaggcccgtagtctgcaaatccttttatgattttctatcaaacaaaagaggaaaatagaccagttgcaatccaaacgagagtctaatagaatgaggtcgaaaagtaaatcgcgcgggtttgttactgataaagcaggcaagacctaaaatgtgtaaagggcaaagtgtatactttggcgtcaccccttacatattttaggtctttttttattgtgcgtaactaacttgccatcttcaaacaggagggctggaagaagcagaccgctaacacagtacataaaaaaggagacatgaacg |
| *P_amp_* | Promoter  (Lee et al., 2011) | cgcggaacccctatttgtttatttttctaaatacattcaaatatgtatccgctcatgagacaataaccctgataaatgcttcaataatattgaaaaaggaagagt |
| *sacb* | Levansucrose gene  (Quant, Hynes, 1993) | atgaacatcaaaaagtttgcaaaacaagcaacagtattaacctttactaccgcactgctggcaggaggcgcaactcaagcgtttgcgaaagaaacgaaccaaaagccatataaggaaacatacggcatttcccatattacacgccatgatatgctgcaaatccctgaacagcaaaaaaatgaaaaatatcaagttcctgaattcgattcgtccacaattaaaaatatctcttctgcaaaaggcctggacgtttgggacagctggccattacaaaacgctgacggcactgtcgcaaactatcgcggctaccacatcgtctttgcattagccggagatcctaaaaatgcggatgacacatcgatttacatgttctatcaaaaagtcggcgaaacttctattgacagctggaaaaacgctggccgcgtctttaaagacagcgacaaattcgatgcaaatgattctatcctaaaagaccaaacacaagaatggtcaggttcagccacatttacatctgacggaaaaatccgtttattctacactgatttctccggtaaacattacggcaaacaaacactgacaactgcacaagttaacgtatcagcatcagacagctctttgaacatcaacggtgtagaggattataaatcaatctttgacggtgacggaaaaacgtatcaaaatgtacagcagttcatcgatgaaggcaactacagctcaggcgacaaccatacgctgagagatcctcactacgtagaagataaaggccacaaatacttagtatttgaagcaaacactggaactgaagatggctaccaaggcgaagaatctttatttaacaaagcatactatggcaaaagcacatcattcttccgtcaagaaagtcaaaaacttctgcaaagcgataaaaaacgcacggctgagttagcaaacggcgctctcggtatgattgagctaaacgatgattacacactgaaaaaagtgatgaaaccgctgattgcatctaacacagtaacagatgaaattgaacgcgcgaacgtctttaaaatgaacggcaaatggtacctgttcactgactcccgcggatcaaaaatgacgattgacggcatttcgtctaacgatatttacatgcttggttatgtttctaattctttaactggcccatacaagccgctgaacaaaactggccttgtgttaaaaatggatcttgatcctaacgatgtaacctttacttactcacacttcgctgtacctcaagcgaaaggaaacaatgtcgtggtgattacaagctatatgacaaacagaggattctacgcagacaaacaatcaacgtttgcgccaagcttcctgctgaacatcaaaggcaagaaaacatctgttgtcaaagacagcatccttgaacaaggacaattaacagttaacaaataa |
| *eyfp* | Fluorescent reporter gene  (Knoot et al., 2019) | atggtgagcaagggcgaggagctgttcaccggggtggtgcccatcctggtcgagctggacggcgacgtaaacggccacaagttcagcgtgtccggcgagggcgagggcgatgccacctacggcaagctgaccctgaagttcatctgcaccaccggcaagctgcccgtgccctggcccaccctcgtgaccaccttcggctacggcctgcaatgcttcgcccgctaccccgaccacatgaagctgcacgacttcttcaagtccgccatgcccgaaggctacgtccaggagcgcaccatcttcttcaaggacgacggcaactacaagacccgcgccgaggtgaagttcgagggcgacaccctggtgaaccgcatcgagctgaagggcatcgacttcaaggaggacggcaacatcctggggcacaagctggagtacaactacaacagccacaacgtctatatcatggccgacaagcagaagaacggcatcaaggtgaacttcaagatccgccacaacatcgaggacggcagcgtgcagctcgccgaccactaccagcagaacacccccatcggcgacggccccgtgctgctgcccgacaaccactacctgagctaccagtccgccctgagcaaagaccccaacgagaagcgcgatcacatggtcctgctggagttcgtgaccgccgccgggatcactctcggcatggacgagctgtacaaggctgcgaattga |
|  | 5’ 0.01 UTR  5’ 200 UTR  5’ 483 UTR (Original)  5’ 1090 UTR  5’ 5000 UTR | ttaggtcgaattcaggtaccatcacctgcatcgttac  gttgttaagtcaataaaggtaccatttggt  tttcaggaattcaaaagatcttttaagaaggagatatacat  ttttcgatcaaacatagtaggaggtctcct  ttaattttgtctggttagattagggggtttttag |
| *mrfp* | Fluorescent reporter gene  (Bi et al., 2013) | atgagtaaaggagaagaacttttcactggagttgtcccaattcttgttgaattagatggtgatgttaatgggcacaaattttctgtcagtggagagggtgaaggtgatgcaacatacggaaaacttacccttaaatttatttgcactactggaaaactacctgttccgtggccaacacttgtcactactttctcttatggtgttcaatgcttttcccgttatccggatcacatgaaacggcatgactttttcaagagtgccatgcccgaaggttatgtacaggaacgcactatatctttcaaagatgacgggaactacaagacgcgtgctgaagtcaagtttgaaggtgatacccttgttaatcgtatcgagttaaaaggtattgattttaaagaagatggaaacattctcggacacaaactggagtacaactataactcacacaatgtatacatcacggcagacaaacaaaagaatggaatcaaagctaacttcaaaattcgccacaacattgaagatggctccgttcaactagcagaccattatcaacaaaatactccaattggcgatggccctgtccttttaccagacaaccattacctgtccacacaatctgccctttcgaaagatcccaacgaaaagcgtgaccacatggtccttcttgagtttgtaactgctgctgggattacacatggcatggatgagctctacaaataa |
| *lacZ* | Gene  E. coli MG1655  Gene ID: 945006^a^ | atgaccatgattacggattcactggccgtcgttttacaacgtcgtgactgggaaaaccctggcgttacccaacttaatcgccttgcagcacatccccctttcgccagctggcgtaatagcgaagaggcccgcaccgatcgcccttcccaacagttgcgcagcctgaatggcgaatggcgctttgcctggtttccggcaccagaagcggtgccggaaagctggctggagtgcgatcttcctgaggccgatactgtcgtcgtcccctcaaactggcagatgcacggttacgatgcgcccatctacaccaacgtgacctatcccattacggtcaatccgccgtttgttcccacggagaatccgacgggttgttactcgctcacatttaatgttgatgaaagctggctacaggaaggccagacgcgaattatttttgatggcgttaactcggcgtttcatctgtggtgcaacgggcgctgggtcggttacggccaggacagtcgtttgccgtctgaatttgacctgagcgcatttttacgcgccggagaaaaccgcctcgcggtgatggtgctgcgctggagtgacggcagttatctggaagatcaggatatgtggcggatgagcggcattttccgtgacgtctcgttgctgcataaaccgactacacaaatcagcgatttccatgttgccactcgctttaatgatgatttcagccgcgctgtactggaggctgaagttcagatgtgcggcgagttgcgtgactacctacgggtaacagtttctttatggcagggtgaaacgcaggtcgccagcggcaccgcgcctttcggcggtgaaattatcgatgagcgtggtggttatgccgatcgcgtcacactacgtctgaacgtcgaaaacccgaaactgtggagcgccgaaatcccgaatctctatcgtgcggtggttgaactgcacaccgccgacggcacgctgattgaagcagaagcctgcgatgtcggtttccgcgaggtgcggattgaaaatggtctgctgctgctgaacggcaagccgttgctgattcgaggcgttaaccgtcacgagcatcatcctctgcatggtcaggtcatggatgagcagacgatggtgcaggatatcctgctgatgaagcagaacaactttaacgccgtgcgctgttcgcattatccgaaccatccgctgtggtacacgctgtgcgaccgctacggcctgtatgtggtggatgaagccaatattgaaacccacggcatggtgccaatgaatcgtctgaccgatgatccgcgctggctaccggcgatgagcgaacgcgtaacgcgaatggtgcagcgcgatcgtaatcacccgagtgtgatcatctggtcgctggggaatgaatcaggccacggcgctaatcacgacgcgctgtatcgctggatcaaatctgtcgatccttcccgcccggtgcagtatgaaggcggcggagccgacaccacggccaccgatattatttgcccgatgtacgcgcgcgtggatgaagaccagcccttcccggctgtgccgaaatggtccatcaaaaaatggctttcgctacctggagagacgcgcccgctgatcctttgcgaatacgcccacgcgatgggtaacagtcttggcggtttcgctaaatactggcaggcgtttcgtcagtatccccgtttacagggcggcttcgtctgggactgggtggatcagtcgctgattaaatatgatgaaaacggcaacccgtggtcggcttacggcggtgattttggcgatacgccgaacgatcgccagttctgtatgaacggtctggtctttgccgaccgcacgccgcatccagcgctgacggaagcaaaacaccagcagcagtttttccagttccgtttatccgggcaaaccatcgaagtgaccagcgaatacctgttccgtcatagcgataacgagctcctgcactggatggtggcgctggatggtaagccgctggcaagcggtgaagtgcctctggatgtcgctccacaaggtaaacagttgattgaactgcctgaactaccgcagccggagagcgccgggcaactctggctcacagtacgcgtagtgcaaccgaacgcgaccgcatggtcagaagccgggcacatcagcgcctggcagcagtggcgtctggcggaaaacctcagtgtgacgctccccgccgcgtcccacgccatcccgcatctgaccaccagcgaaatggatttttgcatcgagctgggtaataagcgttggcaatttaaccgccagtcaggctttctttcacagatgtggattggcgataaaaaacaactgctgacgccgctgcgcgatcagttcacccgtgcaccgctggataacgacattggcgtaagtgaagcgacccgcattgaccctaacgcctgggtcgaacgctggaaggcggcgggccattaccaggccgaagcagcgttgttgcagtgcacggcagatacacttgctgatgcggtgctgattacgaccgctcacgcgtggcagcatcaggggaaaaccttatttatcagccggaaaacctaccggattgatggtagtggtcaaatggcgattaccgttgatgttgaagtggcgagcgatacaccgcatccggcgcggattggcctgaactgccagctggcgcaggtagcagagcgggtaaactggctcggattagggccgcaagaaaactatcccgaccgccttactgccgcctgttttgaccgctgggatctgccattgtcagacatgtataccccgtacgtcttcccgagcgaaaacggtctgcgctgcgggacgcgcgaattgaattatggcccacaccagtggcgcggcgacttccagttcaacatcagccgctacagtcaacagcaactgatggaaaccagccatcgccatctgctgcacgcggaagaaggcacatggctgaatatcgacggtttccatatggggattggtggcgacgactcctggagcccgtcagtatcggcggaattccagctgagcgccggtcgctaccattaccagttggtctggtgtcaaaaataa |
|  | 5’ UTR | aattgtgagcggataacaatttcacacaggaaacagct |
| *gfpuv* | Fluorescent reporter gene  (Lee et al., 2011) | **A**tgagtaaaggagaagaacttttcactggagttgtcccaattcttgttgaattagatggtgatgttaatgggcacaaattttctgtcagtggagagggtgaaggtgatgcaacatacggaaaacttacccttaaatttatttgcactactggaaaactacctgttccgtggccaacacttgtcactactttctcttatggtgttcaatgcttttcccgttatccggatcacatgaaacggcatgactttttcaagagtgccatgcccgaaggttatgtacaggaacgcactatatctttcaaagatgacgggaactacaagacgcgtgctgaagtcaagtttgaaggtgatacccttgttaatcgtatcgagttaaaaggtattgattttaaagaagatggaaacattctcggacacaaactggagtacaactataactcacacaatgtatacatcacggcagacaaacaaaagaatggaatcaaagctaacttcaaaattcgccacaacattgaagatggctccgttcaactagcagaccattatcaacaaaatactccaattggcgatggccctgtccttttaccagacaaccattacctgtccacacaatctgccctttcgaaagatcccaacgaaaagcgtgaccacatggtccttcttgagtttgtaactgctgctgggattacacatggcatggatgagctctacaaataa |
|  | 5’ UTR | aattgtgagcggataacaatttcatttcagaattcaaaagatcttttaagaaggagatatacat |

^a^ http://www.ncbi.nlm.nih.gov/gene

**Supplementary Table III.** Flanking regions used for sucrose counter-selection and double homologous recombination

| **Name** | **DNA sequence** | **Location** |
| --- | --- | --- |
| 5’ HA CAT | gagtccttcttcacatacagcaccgagtgatagcccttggagccgtcaccgttgacctcgatggcgaacggctcgaccttggcgccgaccagataggcgcgggcgtaggacgccgggccgtacatgccgatgtggatgttgccggcgcgctggccttcgatcaccgcggcgtagtcgttggcgatgcggagtttcaccggcacgccgagctgcttggtgaggtagtcggtcagcggggtccagcgggtggtggtgcccgaggcgttctcgtccgggatcttgccgaacaccagttcgggatatttcgccttccagtcctgcgccggcgcggcgtgcgccgtgaacgccagcgcggcagcggcggcgacgagagtacggagcttgatcatgacgtttcctgtctcttcaagtcgatgaacggtcgaaacacggcaaccgcacgcggtcgcgccgcacgcggtgccggataggggagacggcgaacgatcgccgccgggatcaggccacggcgacgccggccaatgcgggggcgggaaggccgtgcggcacgtgcgacgcggtgccgagtacttcgtcggcttcgaggtcgtagagttcgcgcgcgatctggtcggtgagcgcggccggcgcgccatcgaacaccacgcggccggaggccatgccgatcagtcggtcgcaatagctgcgggccagatcgagcgagtgcagattgcacagcacggtgatgccgaagtgcttgttgatgcgcagcagcgcatccatcacgatcttggtgttgcgcggatcgagcgaggcgatcggctcgtcggcgagcacgatatcgggctgctgcaccagggcgcgggcgatcgcgacgcgctgctgctgaccgccggagagctggtcggcgcgctgcgccgcgtaggacgccatgtcgaactgatcgagcgccgagatcgccagggcgcgatcctgctcgggccacatctgcaccagcgagcgccacgacggcacttcggacagccggcccatcagcacgttggtcagcacgtcgagccggccgatcaggttgaattgctggaagatcatcgccgagcgtgcgcgccattgccgcagctcgcggccctgcagcgcggtcacatcaataccttcgaacaggatgcggccctcggaaggctccgccagccggttcagcatgcgcagcagagtcgacttgcctgcgccggatcggccgatcaccccgacgaagctgccgcgctccaccgaaaacgatgcgccatctacggcggctttgctgccaaaacggcaggtcagaccctcaactaccagcatgcgctgctcccgaaacaggttcgggaggaatcgctaacgccgttgttttgcaaccgtgtgacagtcgatgtagttccgccgacatatcaaggtcttcgctacgtcatcgatgcgtcacgacgacgtcatgagtggcaccgatgacaggctcaactccaatcatcgcgcacgagaatcccatg | chromosome |
| 3’ HA CAT | aggaaactgtcagtggacgaattcctcgacagatacgaagctgcggccaaggcgcagccggcggacgcccgtgtggctgtaacggacgagcaggtgatgggatgagcgagatcgcgatcgagggcggccgcacgctggtcggcgacgagattgccgagggctcgctgcagatcgctggcggcgcgatcgcggtggtcggttcggcgagcggtcaggcggcgatccggatcgacgcccgcggcctcttggtgttgcccggcatcgtcgatctgcacggcgatgcgttcgagcggcagatgatgccgcggccgggcgtcgatttcccgatcgacgtggcgctgatcgacagcgaccgccaggcgatcgctaacggtctgacgacggtctttcatgccgtcacctggtcgtgggagcccggcctgcgcagtgccgacaacgctcgccggatgctcgacgcgatcgatctgaccaagccgcggctttccgccgacacccgcattcatctgcgccacgaaacctttaatctcgatgcggtcgacgagatcgcgcaatggatcggcgaccgccgcgtcgatctgttcgccttcaacgatcacatggacacgacgctggcgaacctcgccaagccgcagaagcggaaccggatggtcgagcgcaccggtctgtccgacgacgaattcgaccggctggtcgaacgcatcgcggcgcgtggcgacgaagtgccacaggcaagtcggcgcctcgccgccatcgcacgagaggtgtcgatgccgatgctgtcgcacgatgacgacacgccggcgatacggcagggctaccgtgatctcggcgtcggcatcgccgaattccccaccaccgaggagaccgcacgcgacgcggcgctgcacggcgacttcatcgtgttcggctcgccgaacgtggtgcgcggtggcagccacaccggctggaccaaggcggccgacatgatcgccaagggactgtgctcggtgctggcctccgactactactacccggcgcccctgctcgccgcgttccggctggtgcatgacggcatcctgccgctggagcaggcctggaagttgatctcggagaaccccgcccgcgccgctggtctgtctgaacgcggcacgatcgcgagcggtcagcgcgccgacgtgctgctggtcgacgatcgcgtcgcgctgcggccgcggatcgtcgcggtgatcgccggcggccggctggtgcacgtcgacgatccggagcggttcgtgatcacgcgcaaactgccgcacccggctggcgttgccgcttgatctgacgttatcatcatcgg | chromosome |
| 5’ HA 27480 | ttgttgcggtcattggctcaaattcgatctcaggcccgaatcgtaacgcagcctgatgcttttggcaccaaacccacaaagaatcctgtgcatctgcgtggctatttcgtcgaaggggcagaaaccccgttcgggagcctgctgaatcggcggttaggaggcggcgttcgtctttagcctggaaatcgtcggcgggctgacgttgaaaatcggggcgatctcgcggacgcctcgccctgcggccagcatcgcgcgggcttcgtcttgctgggctggggtgagcttgtagggtcggcccaggctcttgcccttcgccttggcgcgggcgcggccttctgacgtgcgggcgaggatggtggcccgttccagcttggcggcggcgcctagcacggtcagcatgaactcggccagctcggtgctggtatcggcccaaggctcggcgatggatttgaaggttgccccggcctccttcacatcgtgcacgatgttcagcaagtcgcgggtggagcgcgcgaggcggtcgatgcgcgtgaccagcagcacgtcccccgcgtccagggcggcgagggccttcttgagctgggggcgctcggcgctggcgccgctaatgttctcgcgaaacaccttggccgctcctgcggccgtgagctgctcgacttgggcggccgtgtcctggtcactggtggagacgcgcgcgtagccgtaaatcatgccgcattgtgtaactgacttatgaaacggtcaagaggcttgatgcagttggatttttctttgtttcataagttttgacttatgaactaacccggctggggaaagcgggtatggcggtttgttgcccttccgttcatttcacgactttcgtataagattcgggttcctccccacttcatttagtaatagtggccgcgacatgcccaaaatcttgc | Endogenous plasmid  pRPA |
| 3’ HA 27480 | gtttcgcctcatcttcccggaatctaaagccgcgttgcacccttcgcttccgttccctccaatttgcaccgaaccggcggcataaatgctggcggtgctaatcatgcctgtggtcgagatgcccggtccgaccaggacgcccgtcgttccgaaataggtggtgccactcaccccgcctgtggtgaagctgatggtgccaccattccccgccaccacatttgcggaggtgctgacgatgcggtcgccgctgttgctgccggtgatggcgacgccattgacggtgagcgcggtcgcgctgatgttggtggccgtgaggcggctgctggcgttggtccaggtgaggccactgttggcatcgaagctgttgcccgtgttgtactggatatagccggtgctgcccgcggccacggtgctgccgccgcttagcgggttgttccagccgttgctggtgccgttgaaaacgcagaaggtgacggcaacgttctccggccgcaagcatattggcgagcgcctggctctcctggtgagggccaactacgtcgctcgggcacgcagcttggcggcccgccccggcgtgcactggctgacgccaaaggcagccacgcacttccaccctgtctttggtgaggaatgggcgcgcgcggcgagtggcaaggggtttacggataacctgcatatgccgcagcggctgctggactacgccatgggccgccagaagggcagcgccgcgacccgcatgatgttcgtgtttggcagcgcggcaatgttagagcgcgccatgaccaccctgcccgaccgcgacgctcgggtctggcgcgcggtgttcttcaaatccttgccggacttggtagaagattttggcgggacctggttgcaggcaactggcgagcgccggaatccgttcgagcgggtggaacctgcccaagcgacaggagccccatagagacgtttttgggggaggtgccgagcgttcccttgctcgcggcgttttaatcgctgtggtggccgctggggctgttctgtgtcgccttggatcaaaggcgaggtccgcctttgtccatggcagccacttt | Endogenous plasmid  pRPA |
| 5’ HA 24785 | ccgcattgtgtaactgacttatgaaacggtcaagaggcttgatgcagttggatttttctttgtttcataagttttgacttatgaactaacccggctggggaaagcgggtatggcggtttgttgcccttccgttcatttcacgactttcgtataagattcgggttcctccccacttcatttagtaatagtggccgcgacatgcccaaaatcttgcgggaaaccgcacatatcagccggaagctgacgcagaacatctttcgagccttcgactatgcgcgtgcaggtgggtttccactcaacctttacgttgtcatcaacattcgggaaactgacgcctgcgccgcagcttcggcctttgaacgtatccgccacaaatatcgggactggttggcccaccactctcgcaagctcggggtccgcatcccgcctatgtacgtgttcacctttgaggcgccaggccatccgcatgtgaactgggcgttgcgcgtcccgccgcgcctggttgatgagtttcagcggaaactgcccggctgggtagagaaggtgcaggggccactaggtccctttgacatcaacgtgcagccgatagcgccggacggcgcatataaggcgctggccaactacatcgtaaaaggctgtgacccggagtatgtggcgcactttcatctcgcagcccttgccgaacagcatggcccgcagggtgcgttttggggaaggcgggcaggggttagcccgtcgctcaacaaagcagagcgagacgcggccggattcaatccaaaacgccgggaggtgcgcagccgctctcatgaacgggacgccgcgtgaatgctacctgcaaacctgcatatgcccggtcgtggggtttcgcctcatcttcccggaatctaaagccgcgttgcacccttcgcttccgttccctccaatttgcaccgaaccggcggcataaatgctggcggtgctaatcatg | Endogenous plasmid  pRPA |
| 3’ HA 24785 | gacaggagtttagccatgggattagtcagagtccctagccattgctgatgcgagattatttaaactttgccaagggatattgaccagacgggccgtgctaccaagccgttgctgtcgcaagtgtcgcttaacccgcttctgggctatccaatggttattaatcttgtaaacgcttatcctgccctgctcactggcaatgtagaaccgcttatggactctaagccagttcttccagtttggactctccaaactcagagggtacttagtccctgcaccgtgcaagtatccgtcaatcaccgtcggttgaggctccatacctacattgcccctatcaagccagttttagcacgctgggaagcacgataatctactgcgactaccctagggtactcccgtagaaaagcgttatgaagtatgaccggcaaaaaaccacattgaccctatcagagcaaagctagcacggtagggaacgcgataatctactgcaacacctataggtctgttgctgacacgcacgatgacgtatgacccttttagcacggtagggagcgtgataatcttctgcaacacctatatagtattgttgcgatcgcgagcgatggcgt | Endogenous plasmid  pRPA |
| 5’ HA 25310 | gcctggttgatgagtttcagcggaaactgcccggctgggtagagaaggtgcaggggccactaggtccctttgacatcaacgtgcagccgatagcgccggacggcgcatataaggcgctggccaactacatcgtaaaaggctgtgacccggagtatgtggcgcactttcatctcgcagcccttgccgaacagcatggcccgcagggtgcgttttggggaaggcgggcaggggttagcccgtcgctcaacaaagcagagcgagacgcggccggattcaatccaaaacgccgggaggtgcgcagccgctctcatgaacgggacgccgcgtgaatgctacctgcaaacctgcatatgcccggtcgtggggtttcgcctcatcttcccggaatctaaagccgcgttgcacccttcgcttccgttccctccaatttgcaccgaaccggcggcataaatgctggcggtgctaatcatgcctgtggtcgagatgcccggtccgaccaggacgcccgtcgttccgaaataggtggtgccactcaccccgcctgtggtgaagctgatggtgccaccattccccgccaccacatttgcggaggtgctgacgatgcggtcgccgctgttgctgccggtgatggcgacgccattgacggtgagcgcggtcgcgctgatgttggtggccgtgaggcggctgctggcgttggtccaggtgaggccactgttggcatcgaagctgttgcccgtgttgtactggatatagccggtgctgcccgcggccacggtgctgccgccgcttagcgggttgttccagccgttgctggtgccgttgaaaacgcagaaggtgacggcaacgttctccggccgcaagcatattggcgagcgcctggctctcctggtgagggccaactacgtcgctcgggcacgcagcttggcggcccgccccggcgtgcactggctgacgccaaaggcagccacgcacttccaccctgtctttggtgaggaatgggcgcgcgcggcgagtggcaaggggtttacggataacctgcatatgc | Endogenous plasmid  pRPA |
| 3’ HA 25310 | ctttgtccatggcagccactttgcactgcctgagctgcagtcaagggcgcgaagcgccgaagggaacccttgacggcttaggcgagggctgtgcctccccctttttcttcaaatccaattttcagaatctaccgccgaaggcgttttaacaagtttctatagttttaagtaagttttaggggtgaaatttcccttttgaatcaaggtggttagagggtggttttgtccgcgatagacggtctgatgtccgtgatagacggttttttgtccgcgatagacggtctcctgtccgcgatagacgttcgggccaaaagttatccacaggccggaatctctgggtttaagtggctgaaaacatgttgttgcgggcgatagacggcttggccggaattgtccgcgatagacggtcgggccaaaaatccgggccaaaattaattggccgcgagctgaatgacttccggcgtggtcggcatcgcgggccgccgcttcataagcaaaatcggccctcccttcccttgctctagcgccaggtcaaaatctggcatcgggaaagtctgcgtgcgggtcacaaactccttcaggtcgaaaaggaacttgcgcggctcccgtgacgagccgctgcgcttgtgaagttcggcgacggaatatcgcgcctcgtccttccccgccgccttgcgggccagccgatagataaaccggccgaggcccgacgtgatgaggaagtaatccgggtgcagagtgagcaagggcaacgccttgtcggatctcacgacgctcgaataaacccagtcggggatgcggatttcgatctggtccaccttgccggtgttggtggagctgatgatggtgtgttcaccaataaggggccgggtatcaacctggcggcgctttccgcccgcaaggtttgtgatggtgatggaggttttcgataaccggcgcagggcggcttcaatctcttgatattgtcgtccgccgctggcgcggcggctgaacttcaaaagatgcgcggctgtcggccgatacatttttggcggcaggcttgggcgcagacctttggcatcgtcaatg | Endogenous plasmid  pRPA |

**Supplementary Table IV.** Segregation primer sets

| Primer Name | Sequence | Amplicon length (bp) | Strain |
| --- | --- | --- | --- |
| upcm_F | GTAGATGACCTTGGC  GAAGAACTTGTCG | 1959 | Wild Type |
| cmKO_R | CGATCCAGACGTC  ATGGCCGATCTC |  |  |
| cmKO_F | TCAACGACTCGCA  GATCACCTACACC | 1999 | Wild Type |
| downcm_R | CCACAACAGATCGCTTTCGCTGC |  |  |
| upcm_F | GTAGATGACCTTGGCGAAGAACTTGTCG | 3743 | Wild Type |
| downcm_R | CCACAACAGATCGCTTTCGCTGC | 3171 | Δcat |
| 25310 KO_F | CCACAAATATCG  GGACTGGTTGG | 2738 | Wild Type |
| 25310 KO_R | CCTATGAAATGAAAG  ACTGCCTTATTACG | 4539 | Δ25310 |
| 24785 KO_F | GTCGATGCGCGT  GACCAGCAGCAC | 2858 | Wild Type |
| 24785 KO_R | CGTTGCCCTTGC  TCACTCTGCACC | 4480 | Δ 24785 |
| 24780 KO_F | GCTTCAGTGACAAAA  GACCAAGCCGCATG | 3014 | Wild Type |
| 24780 KO_R | GGACAAAACCACCCTC  TAACCACCTTGATTC | 4539 | Δ 24780 |
| 25310_F | GCTGCTGGAC  TACGCCATGG | 353 | Wild Type |
| 25310_R | CCTTTGATCCAA  GGCGACACAG |  |  |
| 24785_F | CGTCGTTCCG  AAATAGGTGG | 492 | Wild Type |
| 24785_R | CCATTCCTCAC  CAAAGACAGG |  |  |
| 24780_F | GCAGAACATCT  TTCGAGCCTTC | 514 | Wild Type |
| 24780_R | CGTCTCGCTC  TGCTTTGTTG |  |  |

**Supplementary Table V.** RBS Library

| Colony number | Sequence | Translation Initiation Rate |
| --- | --- | --- |
| 205 | cttggc | 54 |
| 187 | gcatcg | 72 |
| 202 | tgtccg | 81 |
| 392 | gcaagc | 116 |
| 19 | atttga | 167 |
| 232 | acatac | 174 |
| 277 | agatat | 188 |
| 338 | gtggct | 210 |
| 282 | actggt | 244 |
| 224 | caggc | 270 |
| 21 | tggact | 365 |
| 227 | aggcct | 396 |
| 134 | gggctt | 479 |
| 383 | ggtcga | 517 |
| 46 | gtgtag | 622 |
| 444 | tggagc | 665 |
| 437 | tggagc | 665 |
| 239 | gggtca | 680 |
| 45 | aatgag | 1038 |
| 399 | ggtttc | 1081 |
| 197 | gaggct | 1406 |
| 442 | gagggc | 1603 |
| 279 | atggag | 1613 |
| 382 | gtggag | 1613 |
| 108 | gtggag | 1613 |
| 126 | attagg | 2057 |
| 225 | gggtt | 2127 |
| 32 | aggaga | 3029 |
| 159 | aagagg | 3374 |
| 204 | ggggtt | 3700 |
| 226 | acgagg | 4004 |

**Supplementary Table VI.** RT-qPCR primers

| **Primer Name** | **Sequence** | **Amplicon Length (bp)** | **Primer Concentration (nM)** | **cDNA or gDNA Concentration (ng/μL)** | **cDNA or gDNA** | **Efficiency** | **R^2** |
| --- | --- | --- | --- | --- | --- | --- | --- |
| 16SrRNA_F | GCTTAACACAT  GCAAGTCGAAC | 230 | 40 | 0.05 | cDNA | 98.61 | 0.994 |
| 16SrRNA_R2 | TCATCCTCTCA  GACCAGCTAC |  |  |  |  |  |  |
| qmrfp_F | CGTTTCAAAGTTC  GTATGGAAGGTTC | 188 | 50 | 0.25 | cDNA | 104.89 | 0.996 |
| qmrfp_R | GTGTTTAACGTAAG  CTTTGGAACCGTAC |  |  |  |  |  |  |
| qKan_F | CAGTCATAGCCG  AATAGCCTCTC | 161 | 40 | 0.25 | gDNA | 97.64 | 0.979 |
| qKan_R | CAAAGTAAACTGG  ATGGCTTTCTTGC |  |  |  |  |  |  |
| qGent_F | CAAAGTTAGGTG  GCTCAAGTATGG | 182 | 60 | 0.25 | gDNA | 99.04 | 0.968 |
| qGent_R | CGATGAATGTCTT  ACTACGGAGCAAG |  |  |  |  |  |  |
| dxs_F | CTTCTCGACA  CCATTCGCAC | 169 | 40 | 0.25 | gDNA | 95.94 | 0.999 |
| dxs_R | ACATAGTGCAG  TGCGGTAGTC |  |  |  |  |  |  |
| qgfpuv_F | GAGTAAAGGAGAAG  AACTTTTCACTGGAG | 197 | 50 | 40 | cDNA | Just Visualized | |
| qgfpuv_R | CCATAAGAGAAAGT  AGTGACAAGTGTTG |  |  |  |  |  |  |
| qCAT_F | GCACCATCCTG  AACGAAGTC | 187 | 50 | 40 | cDNA | Just Visualized | |
| qCAT_R | GCCTTCGAAA  TAGGTGCTGG |  |  |  |  |  |  |
| qRepA_F | GCACGAAGAA  CATGAGCTGC | 178 | 50 | 0.25 | gDNA | 94.71 | 0.978 |
| qRepA_R | CGACCTTGAGC  TTGAGGGAAAG |  |  |  |  |  |  |

**Supplementary Table VII.** *R. palustris*’ background fluorescence

| **Fluorescent protein name** | **Ex (nm)** | **Em (nm)** | **R. palustris background fluorescence at Ex and Em** |
| --- | --- | --- | --- |
| Sirius | 355 | 424 | 533,458 |
| FbFP | 450 | 495 | 68,500 |
| GFPuv | 395 | 509 | 107,300 |
| eYFP | 485 | 528 | 77,212 |
| mRFP | 583 | 608 | 12,274 |
| eqFP670 | 605 | 670 | 12,211 |


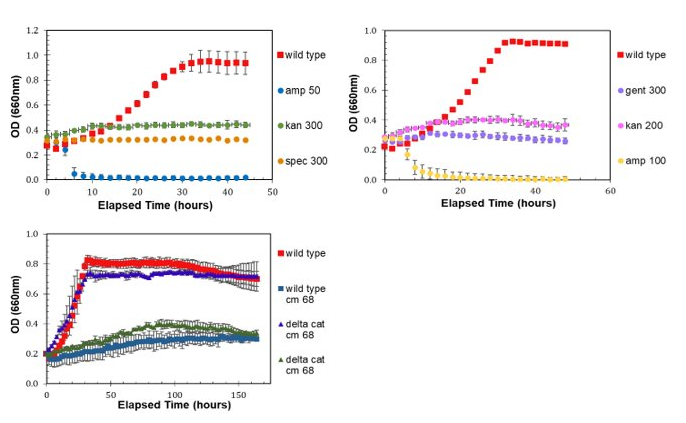


**Supplementary Figure 1.** Growth curve data obtained for antibiotic sensitivity tests. OD_660_ was measured every 2 hours. Error bars represent the population standard deviation.


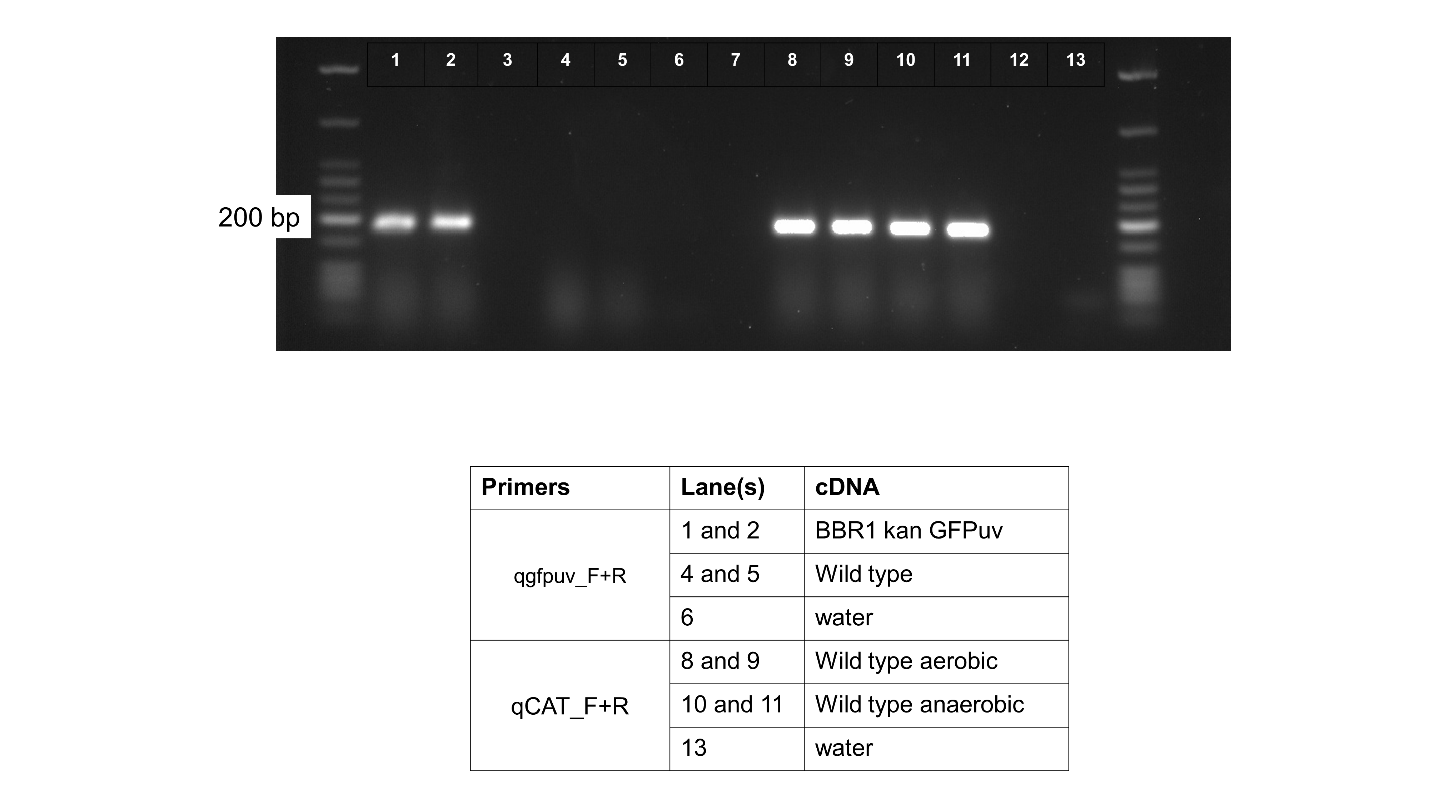


**Supplementary Figure 2.** 2% agarose gel of RT-PCR products showing expression of *gfpuv* in the BBR1 kan GFPuv strain but not the wild type, and expression of *cat* in the wild type strain grown aerobically and anaerobically.


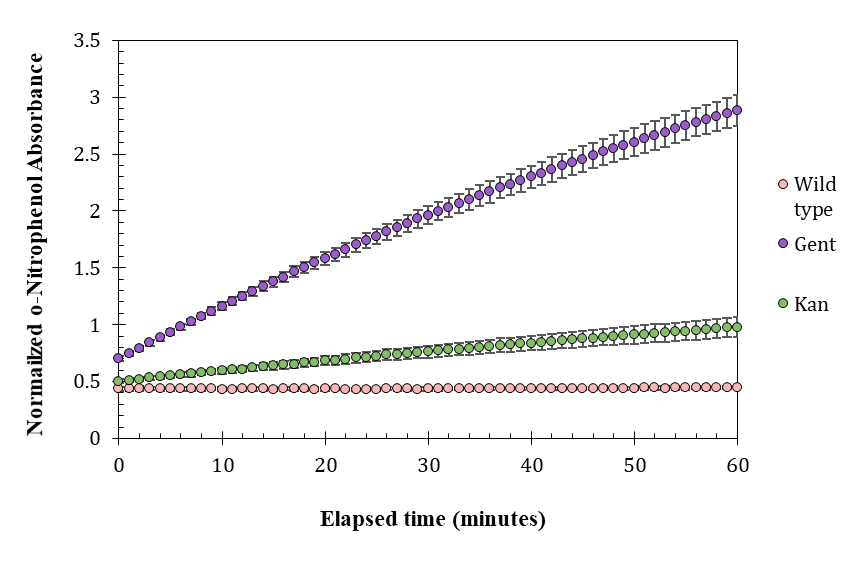


**Supplementary Figure 3.** β-galactosidase assay results for *R. palustris* strains: wild type. BBR1-kan-LacZ, and BBR1-gent-LacZ, grown in triplicate with no antibiotic, 300 µg/mL kanamycin sulfate, or 300 µg/mL gentamicin sulfate respectively added to the media (Materials and Methods). Error bars represent the population standard deviation.


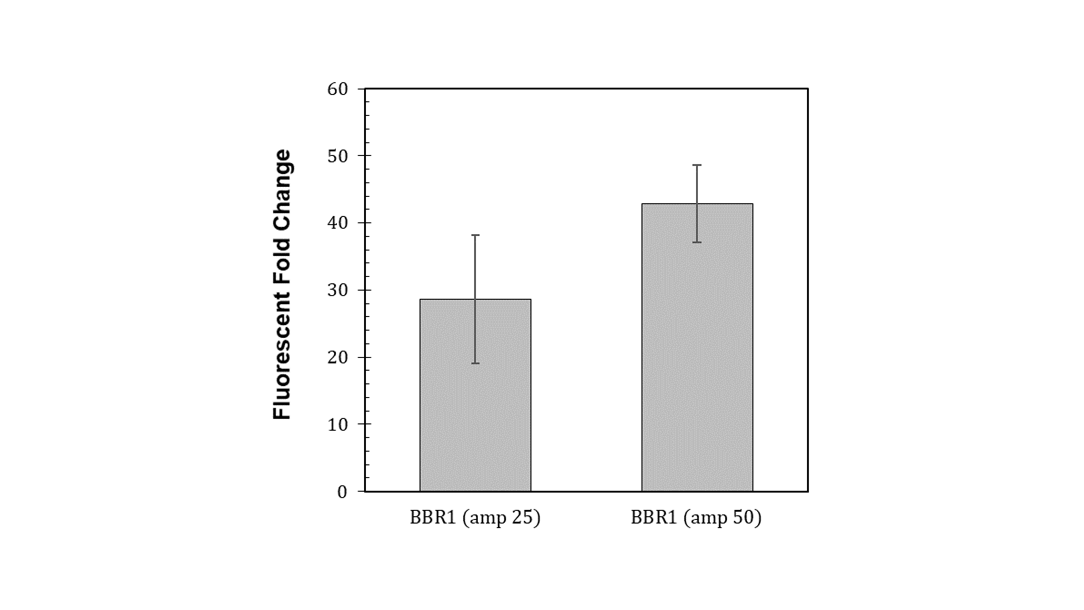


**Supplementary Figure 4.** Fluorescent fold change average of *R. palustris* BBR1 amp mRFP strain grown in triplicate with 25µg/mL or 50 µg/mL ampicillin added to the media (Materials and Methods). Error bars represent the population standard deviation.


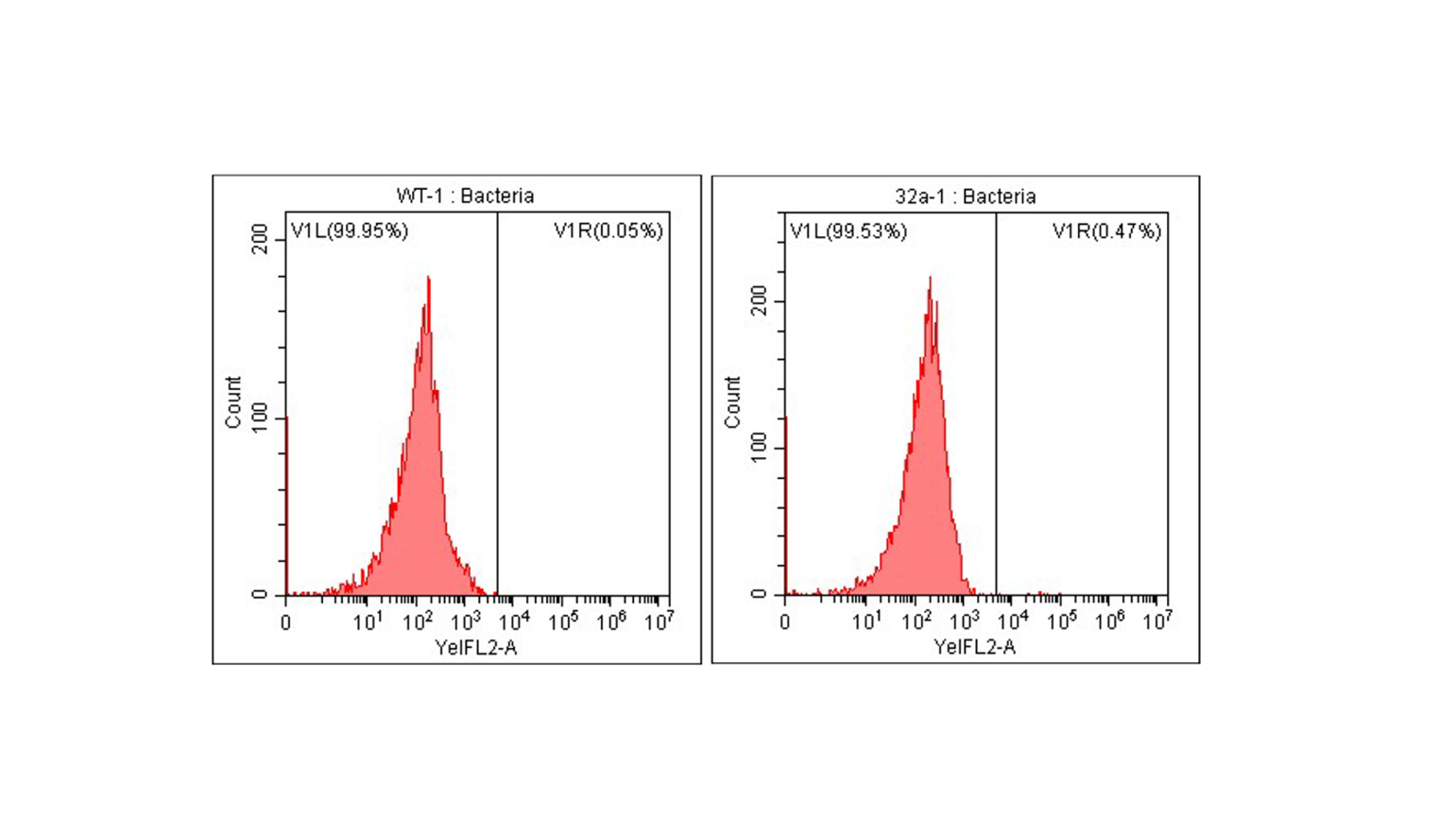
**Supplementary Figure 5.** Representative flow cytometry results of mRFP fluorescence. **A)** Wild type *R. palustris* **B)** *R. palustris* BBR1 amp mRFP strain grown with 100 µg/mL ampicillin added to the media (Materials and Methods).

**A)**

**B)**

**
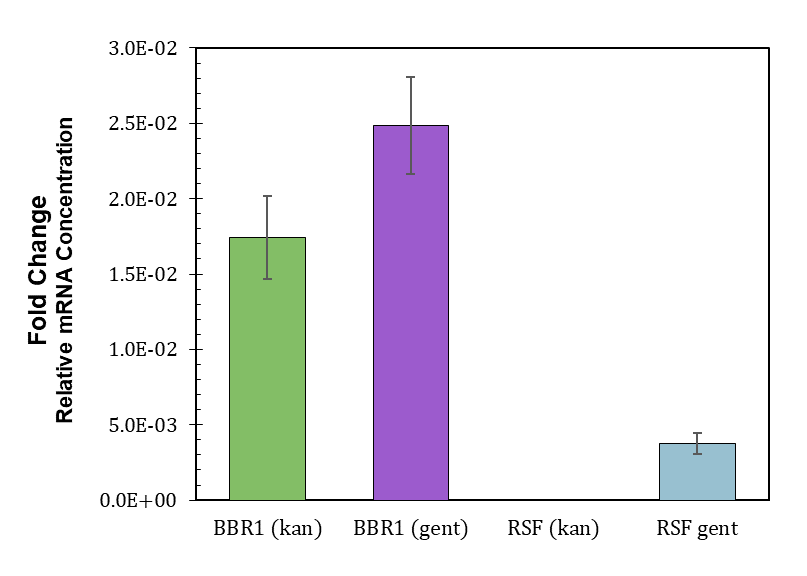
**

**Supplementary Figure 6.** Relative mRNA concentration (*mrfp* relative to *16rRNA*) for the *R. palustris* BBR1 kan, BBR1 gent, RSF1010 kan, and RSF1010 gent strains respectively (Materials and Methods). Two biological and two technical replicates were averaged for each strain. The error bars represent the population standard deviation.

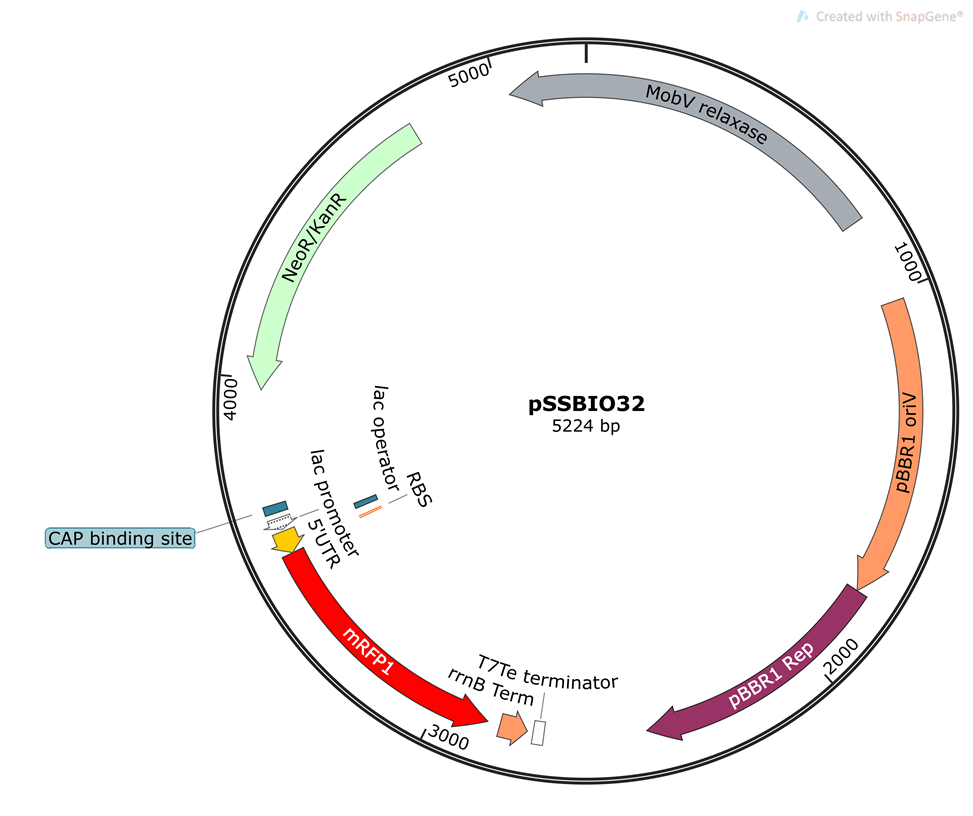


**Supplementary Figure 23.** Map for the BBR1-kan-mRFP plasmid.


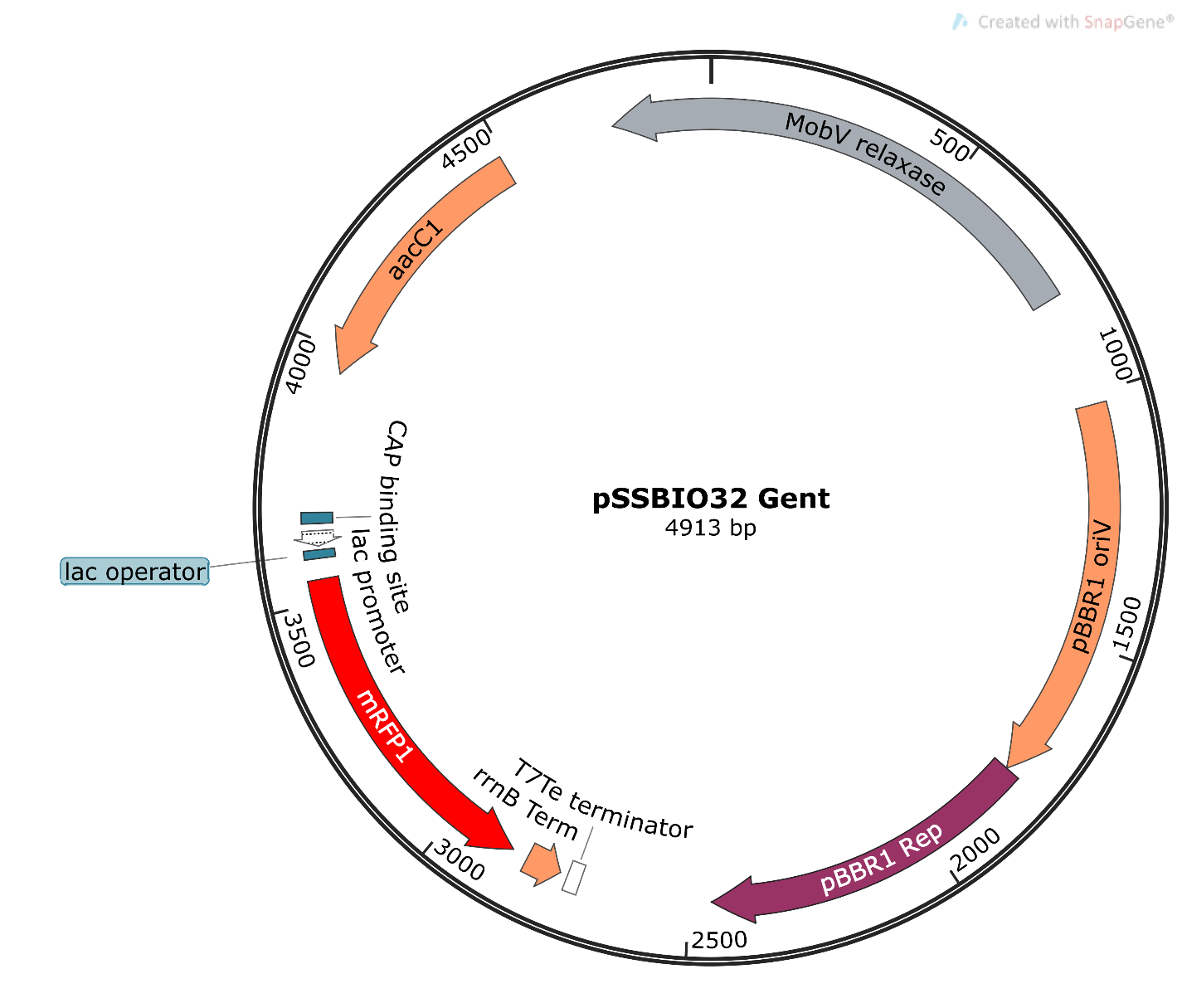


**Supplementary Figure 24.** Map for the BBR1-gent-mRFP plasmid.


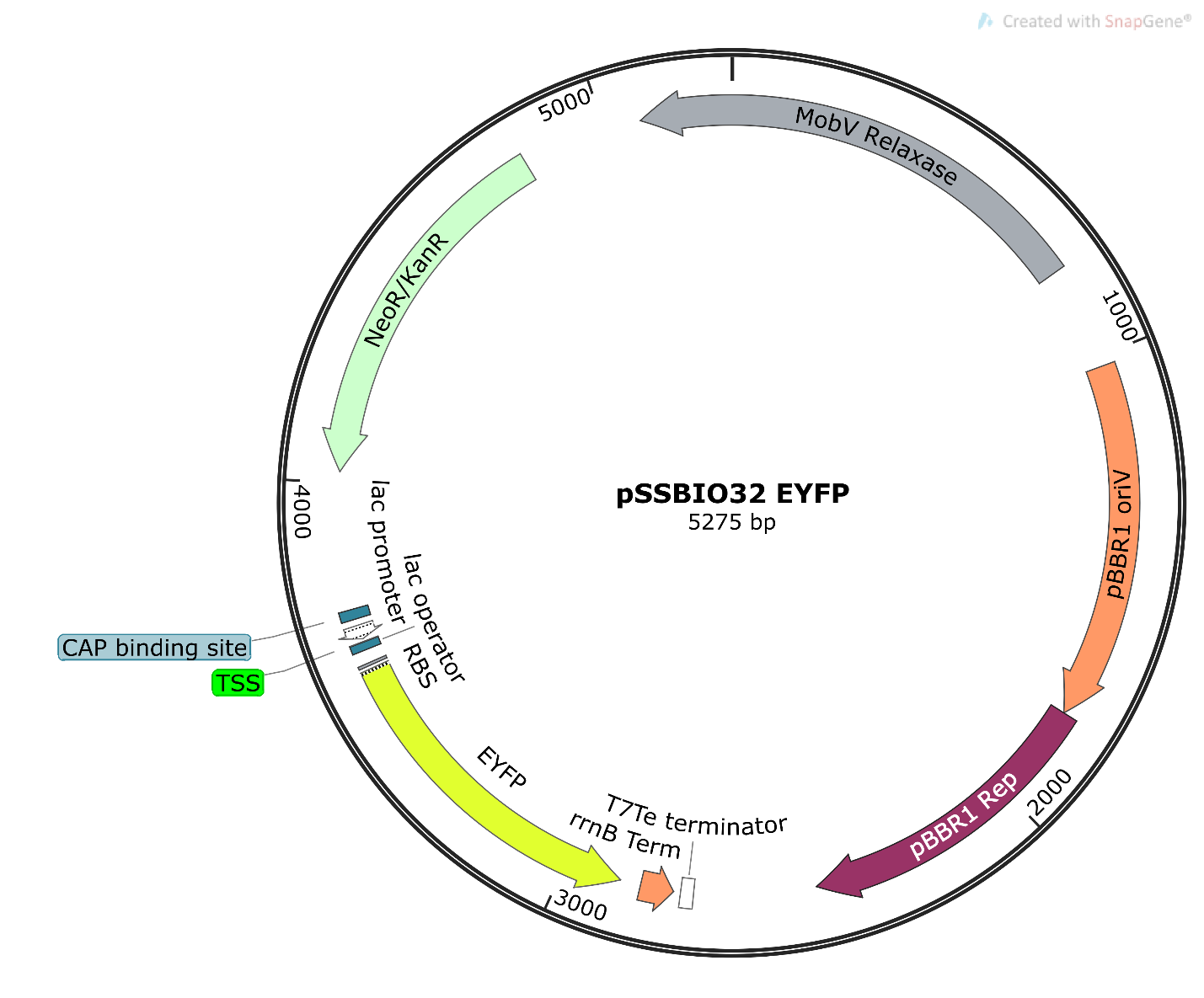


**Supplementary Figure 25.** Map for the BBR1-kan-eYFP plasmid.


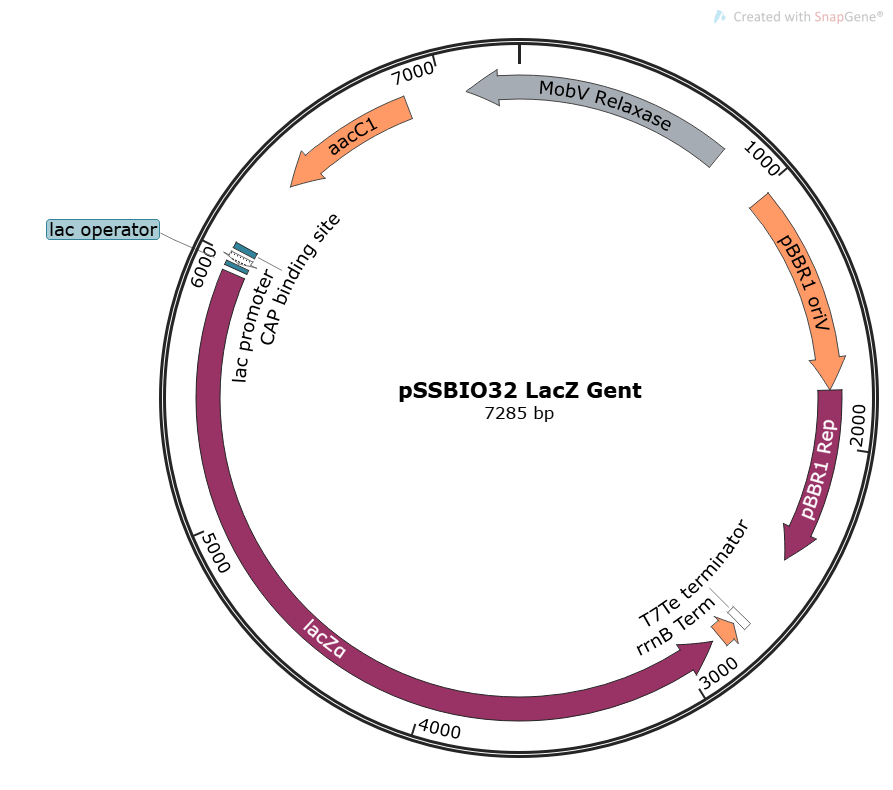


**Supplementary Figure 26.** Map for the BBR1-gent-LacZ plasmid.


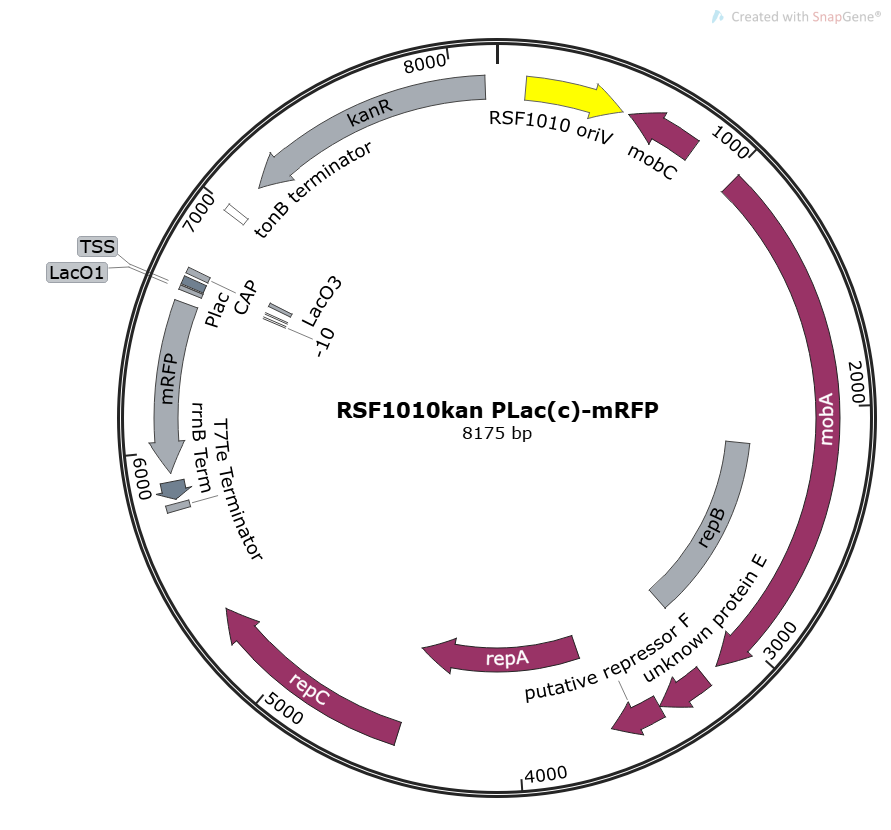


**Supplementary Figure 27.** Map for the RSF1010-kan-mRFP plasmid.

**Reference List**

Bi, C., Su, P., Müller, J., Yeh, Y.C., Chhabra, S.R., Beller, H.R., Singer, S.W., and Hillson, N.J. (2013). Development of a broad-host synthetic biology toolbox for Ralstonia eutropha and its application to engineering hydrocarbon biofuel production. *Microb Cell Fact* 12**,** 107.

Knoot, C.J., Khatri, Y., Hohlman, R.M., Sherman, D.H., and Pakrasi, H.B. (2019). Engineered Production of Hapalindole Alkaloids in the Cyanobacterium Synechococcus sp. UTEX 2973. *ACS Synthetic Biology* 8**,** 1941-1951.

Kovach, M.E., Elzer, P.H., Hill, D.S., Robertson, G.T., Farris, M.A., Roop, R.M., 2nd, and Peterson, K.M. (1995). Four new derivatives of the broad-host-range cloning vector pBBR1MCS, carrying different antibiotic-resistance cassettes. *Gene* 166**,** 175-176.

Lee, T.S., Krupa, R.A., Zhang, F., Hajimorad, M., Holtz, W.J., Prasad, N., Lee, S.K., and Keasling, J.D. (2011). BglBrick vectors and datasheets: A synthetic biology platform for gene expression. *J Biol Eng* 5**,** 12.

Postle, K., and Good, R.F. (1983). DNA sequence of the Escherichia coli tonB gene. *Proc Natl Acad Sci U S A* 80**,** 5235-5239.

Quandt, J., and Hynes, M.F. (1993). Versatile suicide vectors which allow direct selection for gene replacement in gram-negative bacteria. *Gene* 127**,** 15-21.

Young, R.A. (1979). Transcription termination in the Escherichia coli ribosomal RNA operon rrnC. *J Biol Chem* 254**,** 12725-12731.
